# Metabolic Activation and Carcinogenesis of Tobacco-Specific Nitrosamine N’-Nitrosonornicotine (NNN): A Density Function Theory and Molecular Docking Study

**DOI:** 10.3390/ijerph16020178

**Published:** 2019-01-09

**Authors:** Tengjiao Fan, Guohui Sun, Lijiao Zhao, Xin Cui, Rugang Zhong

**Affiliations:** Beijing Key Laboratory of Environmental & Viral Oncology, College of Life Science and Bioengineering, Beijing University of Technology, Beijing 100124, China; fantengjiao2014@emails.bjut.edu.cn (T.F.); sunguohui@bjut.edu.cn (G.S.); cuixin1201@bjut.edu.cn (X.C.); lifesci@bjut.edu.cn (R.Z.)

**Keywords:** N’-nitrosonornicotine, metabolic activation, carcinogenesis, density functional theory, molecular docking

## Abstract

N’-nitrosonornicotine (NNN) is one of the tobacco-specific nitrosamines (TSNAs) that exists widely in smoke and smokeless tobacco products. NNN can induce tumors in various laboratory animal models and has been identified by International Agency for Research on Cancer (IARC) as a human carcinogen. Metabolic activation of NNN is primarily initiated by cytochrome P450 enzymes (CYP450s) via 2′-hydroxylation or 5′-hydroxylation. Subsequently, the hydroxylating intermediates undergo spontaneous decomposition to generate diazohydroxides, which can be further converted to alkyldiazonium ions, followed by attacking DNA to form various DNA damages, such as pyridyloxobutyl (POB)-DNA adducts and pyridyl-N-pyrrolidinyl (py-py)-DNA adducts. If not repaired correctly, these lesions would lead to tumor formation. In the present study, we performed density functional theory (DFT) computations and molecular docking studies to understand the mechanism of metabolic activation and carcinogenesis of NNN. DFT calculations were performed to explore the 2′- or 5′- hydroxylation reaction of (*R*)-NNN and (*S*)-NNN. The results indicated that NNN catalyzed by the ferric porphyrin (Compound I, Cpd I) at the active center of CYP450 included two steps, hydrogen abstraction and rebound reactions. The free energy barriers of the 2′- and 5′-hydroxylation of NNN are 9.82/8.44 kcal/mol (*R*/*S*) and 7.99/9.19 kcal/mol (*R*/*S*), respectively, suggesting that the 2′-(*S*) and 5′-(*R*) pathways have a slight advantage. The free energy barriers of the decomposition occurred at the 2′-position and 5′-position of NNN are 18.04/18.02 kcal/mol (*R*/*S*) and 18.33/19.53 kcal/mol (*R*/*S*), respectively. Moreover, we calculated the alkylation reactions occurred at ten DNA base sites induced by the 2′-hydroxylation product of NNN, generating the free energy barriers ranging from 0.86 to 4.72 kcal/mol, which indicated that these reactions occurred easily. The docking study showed that (*S*)-NNN had better affinity with CYP450s than that of (*R*)-NNN, which was consistent with the experimental results. Overall, the combined results of the DFT calculations and the docking obtained in this study provide an insight into the understanding of the carcinogenesis of NNN and other TSNAs.

## 1. Introduction

**Figure 1:**
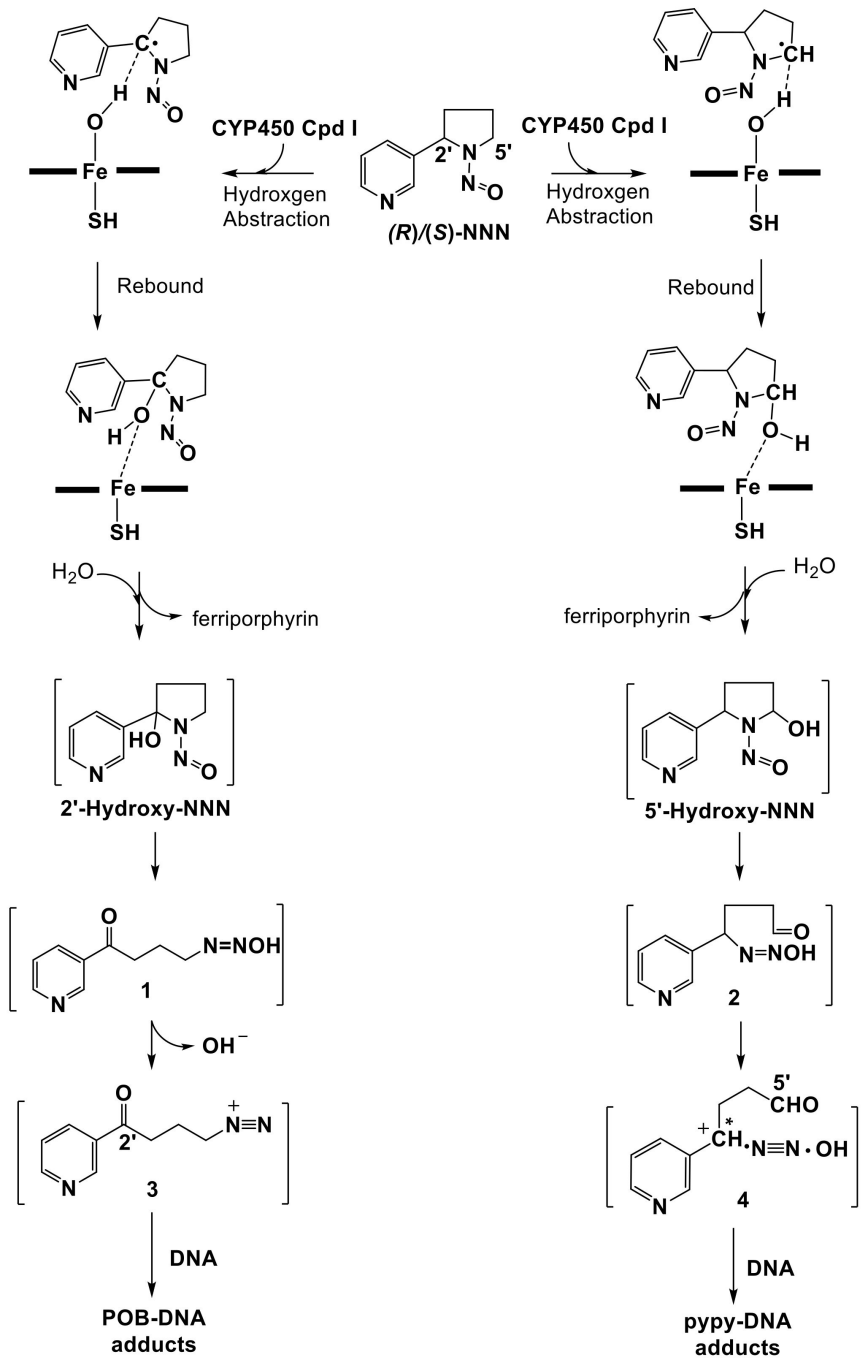
Mechanism of metabolic activation of NNN and the formation of DNA adducts.

N’-nitrosonornicotine (NNN) is one of the tobacco-specific nitrosamines (TSNAs) widely present in tobacco products, especially in smokeless tobacco products [1]. NNN has a chiral center at its 2′-position, thus two enantiomers exist, (*R*)-NNN and (*S*)-NNN, of which (*S*)-NNN is the predominant enantiomer in currently marketed smokeless tobacco products, comprising 57~66% of total NNN [2]. NNN and the related carcinogen 4-(methylnitrosamino)-1-(3-pyridyl)-1-butanone (NNK) have been evaluated by International Agency for Research on Cancer (IARC) as carcinogenic to humans (Group 1) [1]. As a potent carcinogen in animal models, the main carcinogenic target organs of NNN are esophagus, oral cavity, and nasal cavity in rats, lung and forestomach in mice, trachea and nasal cavity in hamsters, and nasal cavity and forebrain in mink [3]. (*S*)-NNN is a powerful oral cavity carcinogen in the male F-344 rat, while (*R*)-NNN has relatively weak carcinogenic activity [4]. NNN requires metabolic activation catalyzed by cytochrome P450 enzymes (CYP450s) to exert its carcinogenic effects [3]. It has two major metabolic pathways, 2′- and 5′-hydroxylation. This leads to the formation of hydroxylating products **1** and **2**, followed by producing the reactive electrophiles **3** and **4**, respectively, which can both react with DNA to produce pyridyloxobutyl (POB)-DNA adducts and pyridyl-N-pyrrolidinyl (py-py)-DNA adducts, respectively (Figure 1). The 2′-hydroxylation pathway was believed to be more mutagenic and carcinogenic than the 5′-hydroxylation in target tissues of rats [5,6,7,8,9,10], while the 5′-hydroxylation of NNN is the more prevalent metabolic pathway in nonhuman primates in vivo and human tissues in vitro [11,12,13,14,15].

The high valent iron-oxo CYP450 species known as compound I (Cpd I) was used to present the CYP450 active site. The proposed mechanism for NNN hydroxylation catalyzed by CYP450 Cpd I involves the initial H-abstraction from a C–H bond of the substrate, followed by the rebound step of the intermediate to form the hydroxylating product [16,17]. Using a computational method to analyze the hydroxylation catalyzed by CYP450s provides a new insight to understand the reaction pathways. Previous studies showed that Cpd I was widely used in related research [18]. The investigations on the hydroxylation of methane [19] and propene [20] mediated by Cpd I using density function theory (DFT) study revealed that H-abstraction reaction was governed by a two-state reactivity (TSR) process, involving high-spin (HS, quartet) and low-spin (LS, doublet) pathways. By using DFT calculations, Takashi et al. [21] explored the mechanistic and energetic aspects for the conversion of camphor to 5-exo-hydroxycamphor by the Cpd I iron-oxo active species. The results indicated that H-abstraction was the rate-determining step in the hydroxylation reaction, while the second transition state of the rebound step that connects the reaction intermediate and the product alcohol complex exhibited a few kcal/mol below that for the H-abstraction on the doublet and quartet potential energy surfaces, which allowed the virtually barrier-free recombination in both spin states, being consistent with experimentally observed high stereo selectivity and short lifetimes of the reaction intermediates [22,23]. Wang et al. [24] studied the reactions of the active species of CYP450 enzyme (Cpd I) with four *para*-(H, Cl, CN, NO_2_) substituted N,N-dimethylanilines (DMA), which indicated that the reaction pathways involved C–H hydroxylation catalyzed by Cpd I and a following nonenzymatic carbinolamine decomposition. It turned out that all of the reactions proceeded in a hydrogen atom transfer (HAT) mechanism, with a mechanistic switch from the spin-selective reactivity for the DMA and p-Cl-DMA substrates to the two-state reactivity for the p-CN-DMA and p-NO_2_-DMA substrates. Ji and coworkers [24] calculated the α-hydroxylation and denitrosation pathways of N-nitrosodimethylamine (NDMA) by taking the six-coordinated triradicaloid oxo-ferryl complex as model for Cpd I that represents the CYP450 active site. The results revealed that the HS pathway predominantly led to detoxification of NDMA by denitrosation, whereas the LS pathway mainly resulted in the α-hydroxylation, followed by dealkylation and subsequent formation of alkyl diazonium ion to exert toxicity.

After metabolic activation mediated by CYP450s, induction of premutant DNA adducts by alkyl diazonium ion is crucial for carcinogenicity of NNN, as shown in Figure 1. Firstly, the α-C atom of NNN is hydroxylated by CYP450 Cpd I to form α-hydroxylated NNN. The α-hydroxylated NNN can spontaneous decomposition to generate diazohydroxide intermediate, followed by converting to alkyl diazonium ions, which attack the DNA base to produce DNA adducts (e.g., POB- and py-py-DNA adducts). These DNA adducts can lead to several types of DNA lesions, such as single/double-stranded breaks (SSBs/DSBs), base modifications, base mismatch, incorporation of bulky adducts during replication, and interstrand/intrastrand crosslinks (ICLs/IALs). Fortunately, this damage can be repaired by DNA repair pathways and restore normal DNA. Some crucial repair factors and the corresponding molecular mechanisms have been reported, which can be used for cancer prevention and therapeutics [25,26,27,28,29,30,31,32]. Two most commonly mentioned pathways to repair DSBs include non-homologous end joining (NHEJ) and homologous recombination (HR). In contrast to NHEJ, HR is an error-free double strand break repair pathway that having important role in preventing tumorigenesis [25,27,29]. Other studies also demonstrated that the Fanconi Anemia (FA) proteins act at different steps of the repair of ICLs in sensing, recognition, and processing of DNA lesions [26,29]. Thus, combination of the precise inhibition of FA pathway with DNA repair inhibitors may further improve the chemotherapeutic efficacy [26,29]. If not repaired correctly, these DNA lesions would lead to the occurrence of tumors. The level of POB-DNA adducts from 2′-hydroxylation of NNN has been quantified by high-performance liquid chromatography electrospray ionization tandem mass spectrometry (HPLC-ESI-MS/MS) in six tissues of rat (oral mucosa, esophageal mucosa, nasal respiratory mucosa, nasal olfactory mucosa, liver, and lung) [10]. Total measured POB-DNA adducts levels in esophageal and oral mucosa were higher in the rats treated with (*S*)-NNN than that of (*R*)-NNN, in which 7-POB-deoxylguanosine (7-POB-dGuo) in the oral cavity and esophagus may play an important role in the carcinogenesis of NNN [10]. In contrast, 2-(2-(3-pyridyl)-*N*-pyrrolidinyl)-2′-deoxyinosine (py-py-dI) was the major DNA adduct resulting from 5′-hydroxylation of NNN in vivo in the rats treated with NNN in the drinking water. The level of py-py-dI adducts in lung and nasal cavity were the highest because the 5′-hydroxylation of NNN is primarily catalyzed by rat CYP450 2A3, which was consistent with the high expression of CYP450 2A3 in these two tissues [33]. 

In the present study, the DFT computation was performed to investigate the metabolic activation of NNN by CYP450 iron-oxo active species Cpd I, the decomposition of 2′- and 5′-hydroxyl-NNN, and the DNA alkylation induced by the active metabolites of NNN. Moreover, a molecular docking study was also carried out to further explain the difference between the carcinogenicity of (*R*)- and (*S*)-NNN and the organ specificity of carcinogenicity. These results are expected to provide theoretical basis for further understanding the carcinogenesis of NNN and other TSNAs.

## 2. Materials and Methods

### 2.1. DFT Calculations

DFT calculations were performed on the CYP450-catalyzed hydroxylation of NNN using the Gaussian 09 program [34] at the unrestricted B3LYP-D3 (UB3LYP-D3) functional [35,36,37] (the standard Becke’s three-parameter exchange potential and the Lee-Yang-Parr correlation functional, Gaussian, Inc., Wallingford, CT, USA) combined with LANL2DZ (Fe)/6-31G (d,p) (H,C,N,O,S) basis set (also called LACVP, here denoted as BSI). We selected the six-coordinated triradicaloid oxo-ferryl complex Fe^4+^O^2−^Por^−^SH^−^ (Por = porphyrin ring = C_20_H_12_N_4_) as a model for Cpd I to represent the CYP450 active site. This thiolate model has been proved that it could match the key axial geometries with available X-ray structure of CYP450 active site [38]. All the structures of the reactants, transition states, intermediates, and products were optimized at the UB3LYP-D3/BSI theoretical level. Geometry optimization was accompanied by the frequency calculations to confirm that all ground states had no imaginary frequency and only one imaginary frequency existed in each transition state. The intrinsic reaction coordinate (IRC) [39,40] calculations were used to confirm that every transition state connected the corresponding reactant and product through the minimized-energy pathway. Considering the effect of the whole protein system on the metabolic reaction, the protein environment was also considered in the energies of single-point calculations. Bulk polarity effects of the protein environment were evaluated with the conductor-like polarizable continuum model (CPCM) [41]. The chlorobenzene (*ε* = 5.6) was chosen as the solvent in the single-point calculations. We selected the LS state of Cpd I to investigate the two hydroxylation pathways of (*R*)- and (*S*)-NNN in our study. The single-point calculations in the chlorobenzene were carried out for the all UB3LYP-D3/BSI-optimized geometries at the CPCM-UB3LYP combined with LANL2DZ (Fe)/6-311++G (2df,2p) (H,C,N,O,S) basis set (here denoted as BSII) to obtain more accurate energies.

In order to better understand the carcinogenic effect of NNN, the decomposition of the α-hydroxylation products and the following alkylation on various DNA base sites by diazonium ions generated from 2′-hydroxylation were also investigated using the DFT computations. We calculated the decomposition process in nonenzymatic environments. The decomposition of the α-hydroxylation products were optimized at the B3LYP/6-31G (d,p) level (here denoted as BSIII) and calculated for single-point at the CPCM-B3LYP/6-311++G (2df,2p) level in water (*ε* = 10.36) (here denoted as BSIV). The reactions of DNA alkylation on various sites were optimized at the B3LYP/BSIII level and single-point calculations were performed at the B3LYP/BSIV level. Methyl substituted purines and pyrimidines were employed as models for DNA bases.

### 2.2. Molecular Docking 

Metabolic experiments have revealed that NNN was catalyzed by different human CYP450s in vitro [42]. Rat CYP450 2A3 is the only known rat CYP450 enzyme being studied for NNN metabolic activation [43]. For our docking study, rat CYP450 2A3 was modeled using the Modeller program [44] due to no corresponding crystal structures available in the Research Collaboration for Structural Bioinformatics (RCSB) Protein Data Bank (PDB) (www.rcsb.org). The amino acid sequence of rat CYP450 2A3 was obtained from the National Center for Biotechnology Information database (NCBI). Identification of candidate templates was performed by the sequence similarity search using basic local alignment search tool (BLAST) search protocol with default values and each target was searched and downloaded from the NCBI database. As a result, the crystal structure with PDB entry 2P85 was used as a template to model rat CYP450 2A3. Easymodeller [45] is a graphical interface to Modeller, which was used to generate the model. The conformational rationality of the modeled CYP450 2A3 was evaluated by the Ramachandran Plot (Figure S1 in the Supplementary Materials).

GOLD (Genetic Optimization for Ligand Docking) Suite 5.2 software was selected for the docking simulations. The geometric structures of (*R*)- and (*S*)-NNN were optimized at the B3LYP/6-31+G(d,p) theoretical level using Gaussian 09 program. Genetic algorithm (GA) of GOLD program was employed for the full range of ligand conformational flexibility as well as the rotational flexibility of selected receptor hydrogen [46,47,48]. The Fe atom of the heme group in CYP450 was chosen as the center for docking and residues within 10 Å around this atom were defined as the binding pocket. In every protein template structure, two criteria were considered: (i) the proximity of the ligand α-C atom toward the heme iron, and (ii) the orientation of the N atom of pyridine. According to published literatures [42], we selected human CYP450 1A1, 2A6, 2A13, 2E1, 3A4 and modeled rat CYP 450 2A3 as receptors and (*R*)- and (*S*)-NNN as ligands. The PDB codes are 1A1 (4I8V, 2.6 Å resolution) [49], 2A6 (4EJJ, 2.3 Å resolution), 2A13 (4EJH, 2.5 Å resolution) [50], 2E1 (3LC4, 3.1 Å resolution) [51], and 3A4 (1W0E, 2.8 Å resolution), respectively [52]. We found that CYP450 1A1, 2A6, 2A13, and 2E1 have more than one similar subchains, and their docking results were analogical, so only chain A was considered for docking. In order to validate the docking approach, a self-docking was conducted using human CYP450 2A13 with PDB entry 4EJH containing nicotine as the protein–ligand crystal complex model. For self-docking, solvent molecules were deleted in the crystal structure of 4EJH, and the ligand nicotine was redocked into the active pocket of 4EJH. Hydrogen atoms were added to the protein and CHEMPLP was chosen as a scoring function. The docking was performed by the “cytochrome P450 mode” in GOLD software.

## 3. Results and Discussion

### 3.1. Metabolic Activation of (R)-NNN and (S)-NNN

**Scheme 1:**
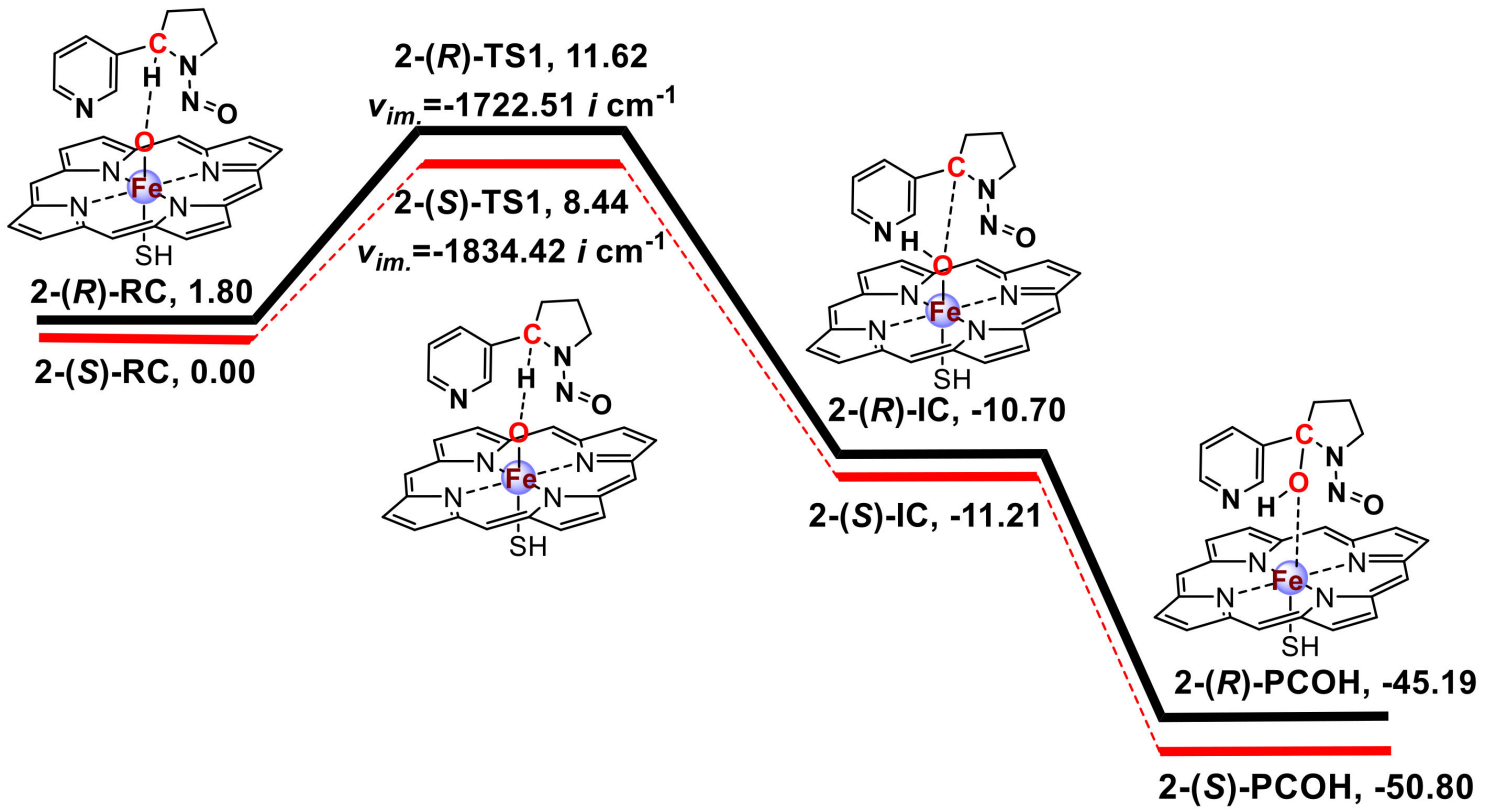
Free energy profile for the 2′-hydroxylation of (*R*)-NNN and (*S*)-NNN by CYP450 Cpd I ^a^. ^a^ Free energy [kcal/mol] at 298.15 K were given related to the reactant complex at the UB3LYP/BSII//BSI level with zero-point correction including solvation (CPCM/BSII, ε = 5.6). RC = reactant complex, TS1 = transition state of H-abstraction, IC = intermediate complex with H abstracted by Cpd I, PCOH = hydroxylation product + Fe (Por SH). 2-(*S*)-RC was used as benchmark (0.00 kcal/mol), and the relative energies of other stationary points were calculated by subtracting the absolute energy of 2-(*S*)-RC.

**Scheme 2:**
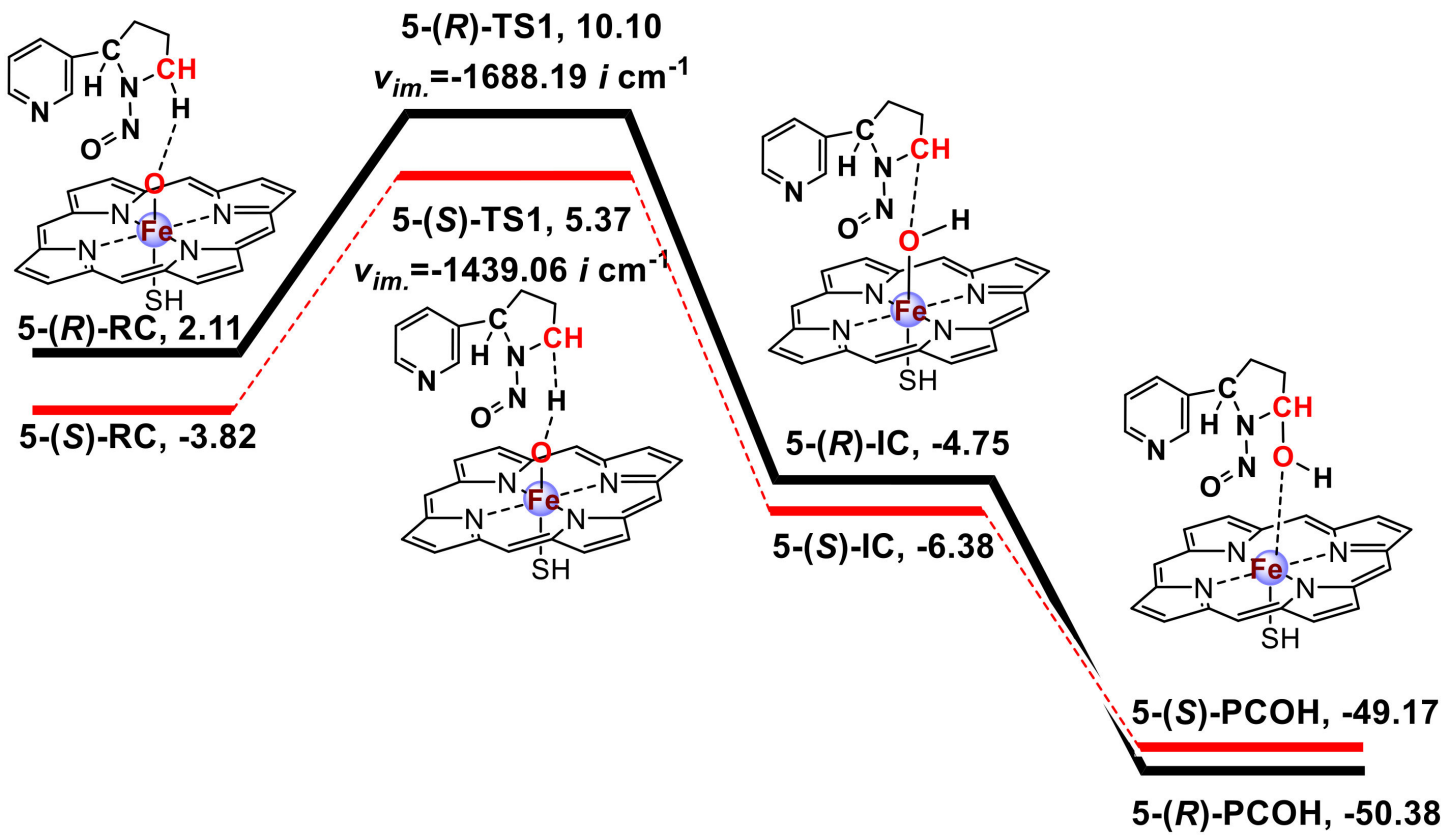
Free energy profile for the 5′-hydroxylation of (*R*)-NNN and (*S*)-NNN by CYP450 Cpd I ^a^. ^a^ Free energy [kcal/mol] at 298.15 K were given related to the reactant complex at the B3LYP/BSII//BSI level with zero-point correction including solvation (CPCM/BSII, ε = 5.6). RC = reactant complex, TS1 = transition state of H-abstraction, IC = intermediate complex with H abstracted by Cpd I, PCOH = hydroxylation product + Fe (Por SH). 2-(*S*)-RC was used as benchmark (0.00 kcal/mol), and the relative energies of other stationary points were calculated by subtracting the absolute energy of 2-(*S*)-RC.

The energy profiles obtained from the calculations of the 2′ and 5′-hydroxylation pathways of NNN at the UB3LYP-D3 BSII//BSI level with LS were plotted in Scheme 1 and Scheme 2, respectively. The absolute energies (AE: a.u.) and relative energies (RE: kcal/mol) for the 2′- and 5′-hydroxylation of *(R)*-NNN and *(S)*-NNN are shown in Table S1 in the Supplementary Materials. The spin densities for the reaction species were calculated at UB3LYP-D3/BSI level (Table S2 in the Supplementary Materials). The coordinate files of four transition states were also shown in the Supplementary Materials. These two schemes informed about the structure of each stationary point and the free energy of the reaction. The energy was corrected with the zero-point energy and then converted to the Gibbs free energy at 298.15 K and 101.325 kPa. The imaginary frequency of each transition state was also marked in these schemes. NNN and Cpd I form reactant complexes (RC) stabilized through a weak hydrogen bond interaction between the Fe-coordinated oxygen and the H atom of the α-carbon of NNN. H-abstraction transfer of the H atom from C–H bond of NNN to the Fe=O moiety of Cpd I produces the intermediate complex (IC) by undergoing a transition state (TS), which is the rate-limiting step for the hydroxylation pathway [53]. After this reaction, the IC produces the hydroxylation product complex (PCOH) immediately. 

A previous study [54] explored the effect of increasing bulk polarity on the H-abstraction of NDMA by CYP450 Cpd I with the *ε* value ranging from 2 to 40 for protein environments. The nonpolar solvent chlorobenzene (*ε* = 5.6) was chosen to simulate bulk protein environment because it gave the lowest free energy compared to the dichloroethane (*ε* = 10.36) and acetonitrile (*ε* = 36.64). In the above study [54], NNN was also calculated in HS and LS states. The result indicated that the HS energy barrier was higher than that of the LS energy barrier, and the HS rebound free energy barrier of NNN was much lower than that of NDMA, which account for the feasibility that NNN could undergo direct denitrosation to generate nornicotine [8]. Furthermore, the theoretical investigations of small alkene oxidation supported that the different spin states of Cpd I preferentially catalyzed distinct chemistry, i.e., the doublet state was associated with substrate hydroxylation, while the quartet state was associated with rearranged products, such as epoxides [20,55,56,57,58,59]. We also calculated the HS energy barriers of the 2′- and 5′-hydroxylation pathways of (*R*)-NNN and (*S*)-NNN, which was 8.02/8.46 kcal/mol (*R*/*S*) for the 2′-hydroxylation and 8.12/10.35 kcal/mol (*R*/*S*) for the 5′-hydroxylation. In general, the energy barriers of the HS pathway were slightly higher than that of the LS pathway except for the 2′-(*R*)-pathway (Table S1 in the Supplementary Materials). Our study complements a recent study by Ma et al. [60]. In that study, all stationary points were calculated in the HS and LS pathways. They calculated the possible NNN activation pathways including α-hydroxylation, β-hydroxylation, pyridine N-oxidation and norcotinine formation without distinguishing the NNN enantiomers. Compared to their publication, our study involved the metabolic activation of (*R*)-NNN and (*S*)-NNN with α-hydroxylation pathways, and performed the docking with human P450 enzymes to gain further insight the carcinogenesis of NNN.

The 2′- and 5′-hydroxylation of NNN have similar reaction process, and the difference of energy barriers was not more than 2 kcal/mol. This result was also supported by Ma et al. (C_2_ LS/HS barriers 15.7/15.4 kcal/mol vs. C_5_ LS/HS barriers 14.2/13.4 kcal/mol) [60]. As shown in Scheme 1 and Scheme 2, we found that the free energy barriers of the 2′- and 5′-hydroxylation of NNN were 9.82/8.44 kcal/mol (*R*/*S*) and 7.99/9.19 kcal/mol (*R*/*S*), respectively, in which the 2′-(*S*)-pathway and 5′-(*R*)-pathway energy barriers were lower than other two pathways and should be advantageous. Furthermore, we found that the energy of (*S*)-RC was lower than (*R*)-RC regardless of in 2′- or 5′-hydrxylation pathways. It seemed that the initial reactant system of (*S*)-NNN was more stable than that of (*R*)-NNN. Moreover, the relative energies of PCOHs derived from 2′-(*S*)-pathway and 5′-(*R*)-pathway were also lower than other two pathways, indicating that 2′-(*S*)- and 5′-(*R*)-PCOHs were more stable than their counterparts. Previous findings indicated that without the effect of hepatic clearance, the formation of NNN-induced DNA adducts would be dependent on the direct activation of NNN after local exposure, because esophagus and oral mucosa directly contacted the carcinogen when NNN was administered in the drinking water [8,9]. The cultured rat esophagus preferentially metabolized (*S*)-NNN through the 2′-hydroxylation and (*R*)-NNN preferentially through the 5′-hydroxylation [8]. To some extent, the results obtained from the quantum chemistry calculation in the present study were consistent with the experimental observation of NNN metabolism in rat esophagus.

In the initial study, we performed the theoretical investigation of hydroxylation reactions by B3LYP method without consideration of the dispersion effects. Afterwards, considering the fact that inclusion of dispersion has a significant effect on the energies and geometries of transition states and encounter complexes in CYP450 Cpd I reaction system [61], B3LYP-D3 method was utilized to optimize the geometry structures, which could effectively improve the description of weak interactions [62]. As expected, we found that there was an obvious difference on the geometry structure and energy between B3LYP and B3LYP-D3 calculations (Figure S2 in the Supplementary Materials). The distance between NNN and Cpd I was farther for the RC optimized by B3LYP method when compared to that of B3LYP-D3 method. Thus, we selected B3LYP-D3 method to calculate the CYP450 Cpd I complex reaction system.

**Figure 2:**
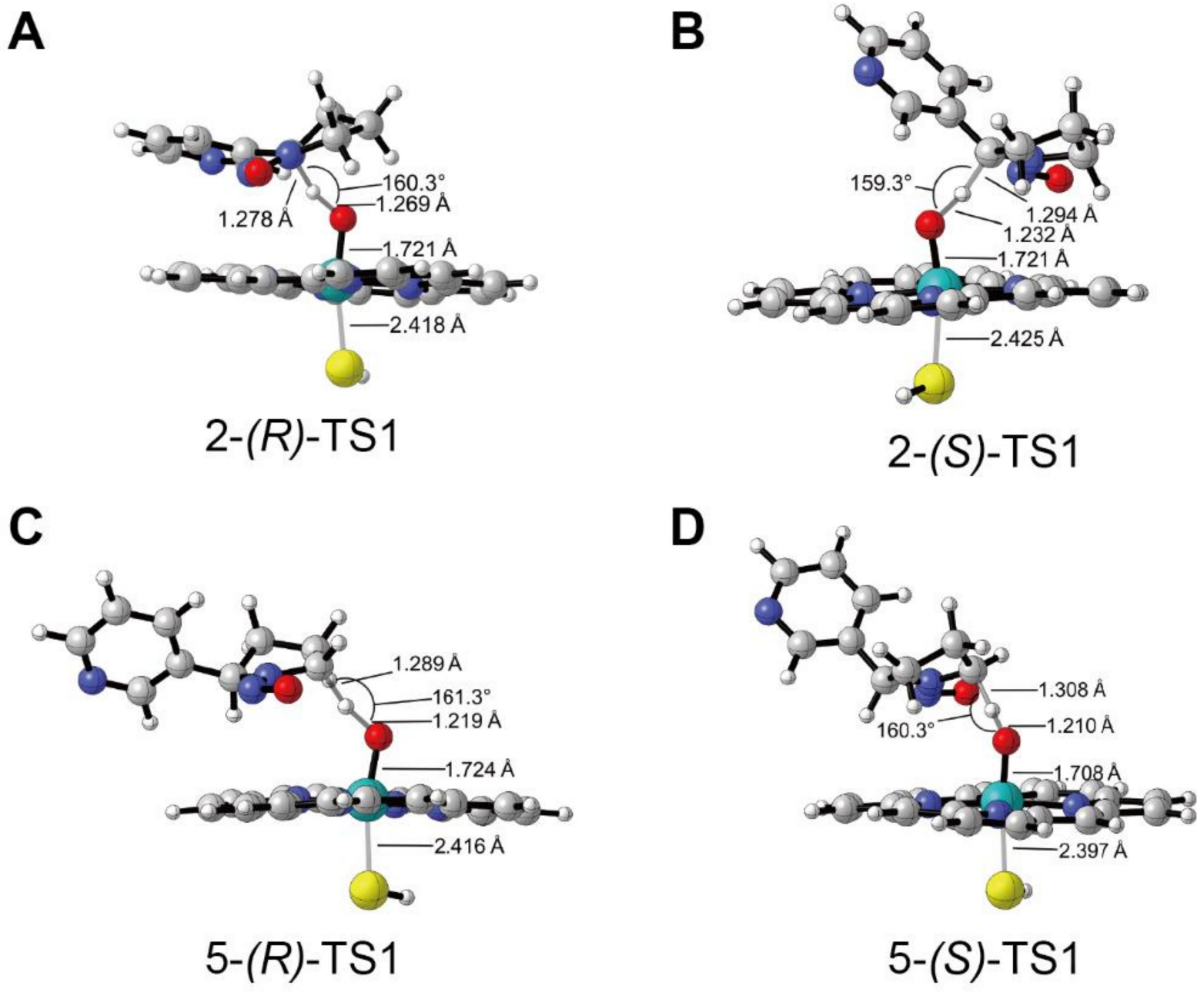
Optimized geometries and main parameters of the transition states of H-abstraction reactions for the 2′- and 5′-hydroxylation of (*R*)-NNN and (*S*)-NNN catalyzed by CYP450 CpdI. (**A**) Transition sate of 2′-hydroxylation of (*R*)-NNN; (**B**) Transition sate of 2′-hydroxylation of (*S*)-NNN; (**C**) Transition sate of 5′-hydroxylation of (*R*)-NNN; (**D**) Transition sate of 5′-hydroxylation of (*S*)-NNN (distances in angstroms). The red atom represents oxygen, the cyan atom represents Fe, the sliver atom represents hydrogen, and the gray atom represents carbon.

**Figure 3:**
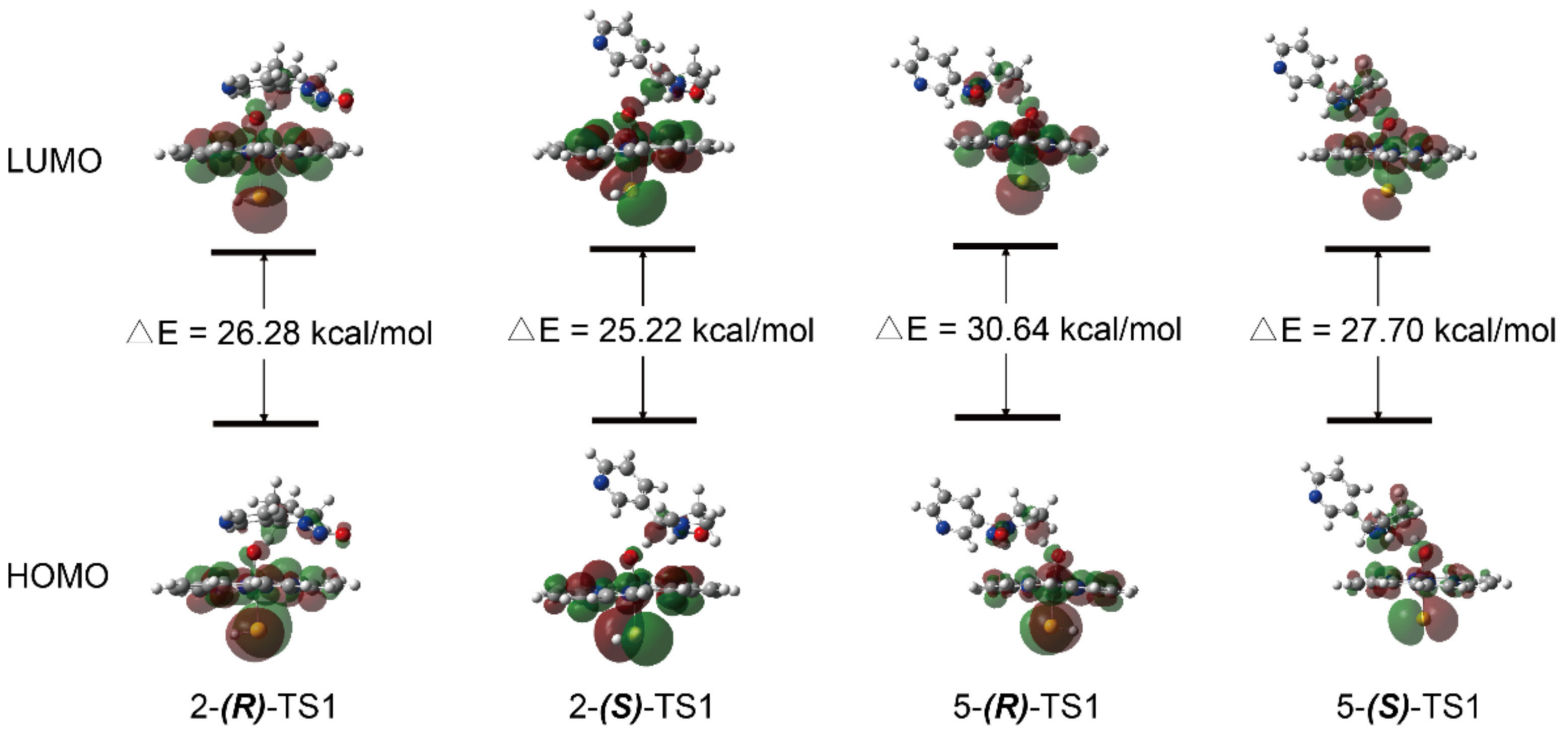
Frontier molecular orbital diagram of four transition states of (*R*)-NNN and (*S*)-NNN catalyzed by CYP450 Cpd I through 2′- and 5′-hydroxylation.

Figure 2 presented the geometries and main parameters of the transition states of H-abstraction reactions for the 2′- and 5′-hydroxylation of (*R*)- and (*S*)-NNN. All optimized geometries for the 2′- and 5′-hydroxylation of (*R*)- and (*S*)-NNN catalyzed by Cpd I on LS states were shown in Figure S3 in the Supplementary Materials. As shown in Figure 2A, the H···O bond distance in 2-(*R*)-TS was 1.269 Å, which was longer than the H···O bond distance (1.232 Å) in 2-(*S*)-TS (Figure 2B), while the C···H bond distance of 2-(*R*)-TS was shorter than 2-*(S)*-TS (1.278 Å vs. 1.294 Å). The angle of C···H···O was 160.3° in 2-(*R*)-TS and 159.3° in 2-(*S*)-TS. The hydrogen atom in 2-(*S*)-TS was placed in close proximity to the oxygen on Fe atom, leading to the favorable formation of the intermediate 2-(*S*)-IC. The H···O bond distances of 5-(*R*)-TS (Figure 2C) and 5-(*S*)-TS (Figure 2D) were 1.219 Å vs. 1.210 Å, and the angle of C···H···O were 161.3° vs. 160.3°. The H···O bond distances were slightly shorter with *S* configuration than that of *R* configuration. Meanwhile, the angle of C···H···O was a little bit smaller with the *S* configuration than that of the *R* configuration. The molecular orbitals of four transition states were shown in Figure 3, which provided some insight into the nature of the transition states. The highest occupied molecular orbital (HOMO) and lowest unoccupied molecular orbital (LUMO) were around the atom C and heme atom O, which indicated the charge transfer from the substrate to Cpd I. The gap Δ*E*_LUMO-HOMO_ of 2-(*S*)-TS1 was 25.22 kcal/mol, which was lower than other transition states, suggesting that the H-abstraction reaction of 2′-hydroxylation of (*S*)-NNN had high reaction activity.

### 3.2. Decomposition of the Hydroxylation Products

**Scheme 3:**
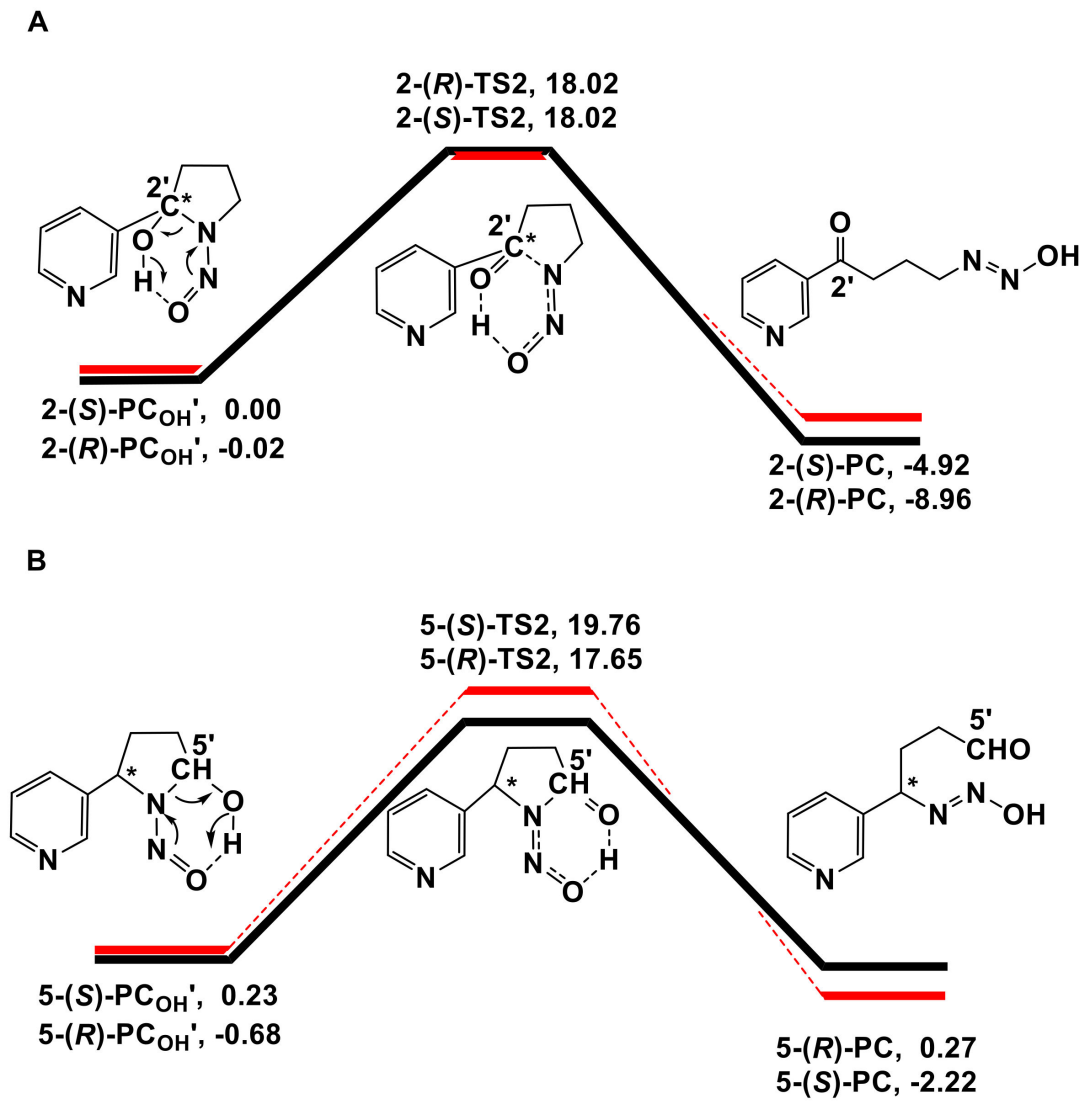
Free energy profiles of the decomposition of 2′- and 5′-hydroxylation products of (*R*)- and (*S*)-NNN ^a^. ^a^ Free energy profiles for the decomposition of 2′-hydroxylation products (**A**) and 5′-hydroxylation products (**B**) of (*R*)-NNN and (*S*)-NNN calculated at the CPCM-B3LYP/6-311++g(2df,2p) level. PC_OH_’ = hydroxylation product; TS2 = transition state of decomposition reaction; PC = diazohydroxide.

**Figure 4:**
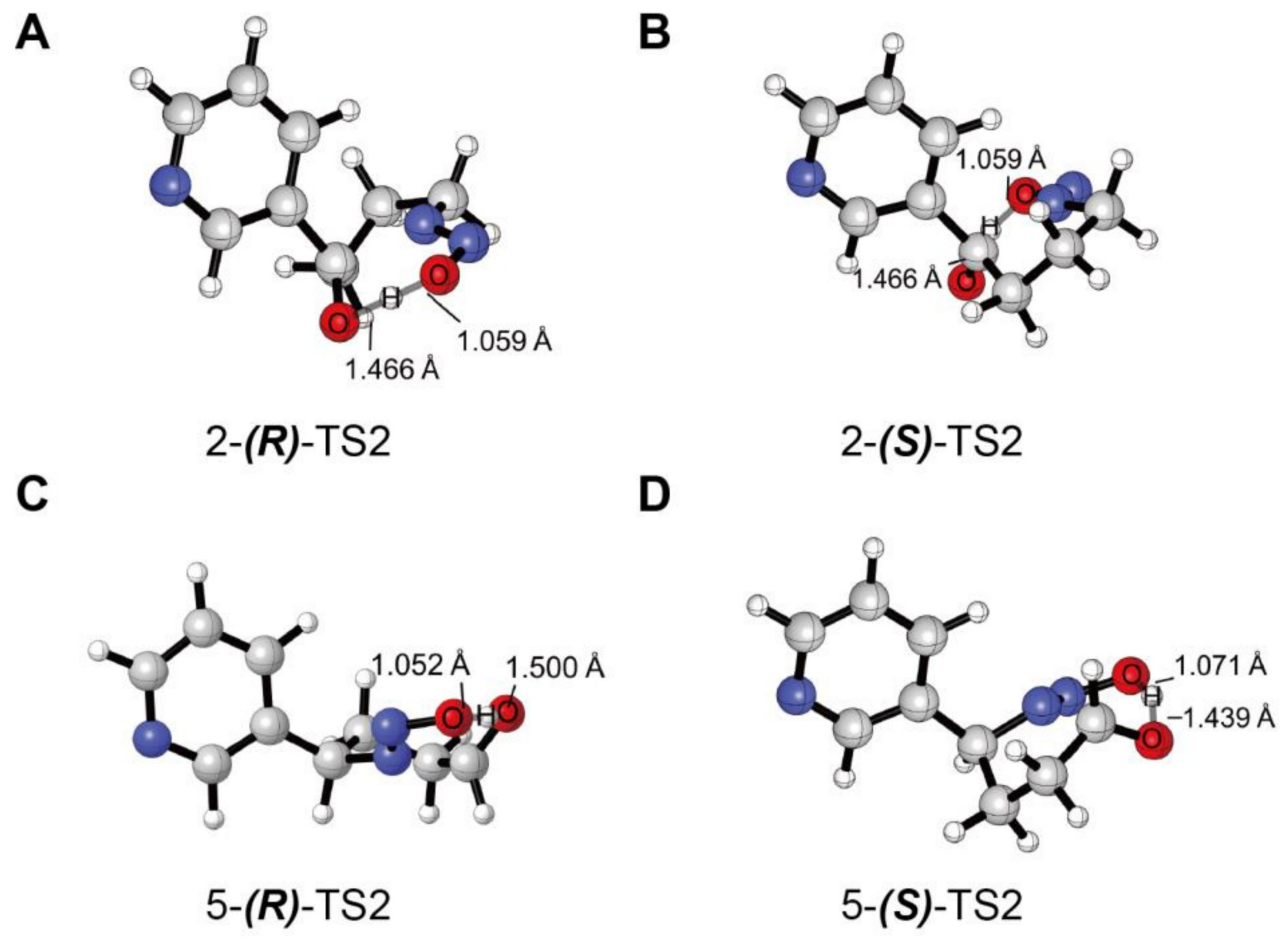
Optimized geometries and main parameters for the transition states of decomposition of (*R*)-NNN and (*S*)-NNN. Transition states of the decomposition reactions of (*R*)-NNN (**A**) and (*S*)-NNN (**B**) via 2′-hydroxylation pathway; Transition states of the decomposition reactions of (*R*)-NNN (**C**) and (*S*)-NNN (**D**) via 5′-hydroxylation pathway (distance in angstroms).

The energy profiles obtained from the decomposition of the hydroxylation products calculated at the B3LYP BSIV/BSIII computational level were plotted in Scheme 3. The absolute energies (AE: a.u.) and relative energies (RE: kcal/mol) for the decomposition of the hydroxylation products were shown in Table S3 in the Supplementary Materials. Transition state coordinate files were also shown in the Supplementary Materials. A previous study indicated that the reaction of C-H hydroxylation of DMA was accompanied by a nonenzymatic carbinolamine decomposition [22]. The generated carbinolaniline could readily dissociate from the heme and decompose in a nonenzymatic environment. The energy barrier of decomposition in the enzymatic environments was 31.5 (28.5) kcal/mol for the quartet (doublet) route and 14.8 kcal/mol in nonenzymatic environments, respectively [22]. According to the above study, we calculated the reaction of decomposition in nonenzymatic environments. The binding energy of hydroxylation products to the heme was quite low (below 5 kcal/mol), so the hydroxylation products could readily dissociate from the heme and be released outside of the enzyme’s pocket. As seen in Scheme 3, the hydroxylation product PC_OH_’ undergoes a transition state (TS2) to form diazohydroxide (PC), which can be further converted to a diazonium ion that directly alkylates DNA bases. The free energy barriers of 2-(*R*)-TS2 and 2-(*S*)-TS2 were almost the same (18.04 vs. 18.02 kcal/mol), while the 5-(*R*)-TS2 was slightly lower than that of the 5-(*S*)-TS2 (18.33 vs. 19.53 kcal/mol). Interestingly, the energy of decomposition product diazohydroxide 5-(*R*)-PC was higher than the corresponding reactant 5-(*R*)-PC_OH_’. However, this would not affect the following alkylation on DNA bases induced by 5′-pathway decomposition products. The diazohydroxide formed by the 5′-pathway will quickly transform into nitrogen ion pair that directly alkylates DNA bases (Figure 1) [63]. The TS2 geometry structures are illustrated in Figure 4. The optimized geometries for the decomposition of the hydroxylation products of (*R*)- and (*S*)-NNN were shown in Figure S4 in the Supplementary Materials. We compared the bond length of O(C)···H and O(N)···H in four TS2, in which the O(N)···H bond length ranged from 1.052 to 1.071 Å, and the O(C)···H bond length ranged from 1.439 to 1.500 Å. These results indicated that there were no significant differences between the decomposition of the 2′- and 5′-hydroxylation products of *(R*) and (*S*)-NNN.

### 3.3. DNA Alkylation of 2′-Hydroxylation Products

**Figure 5:**
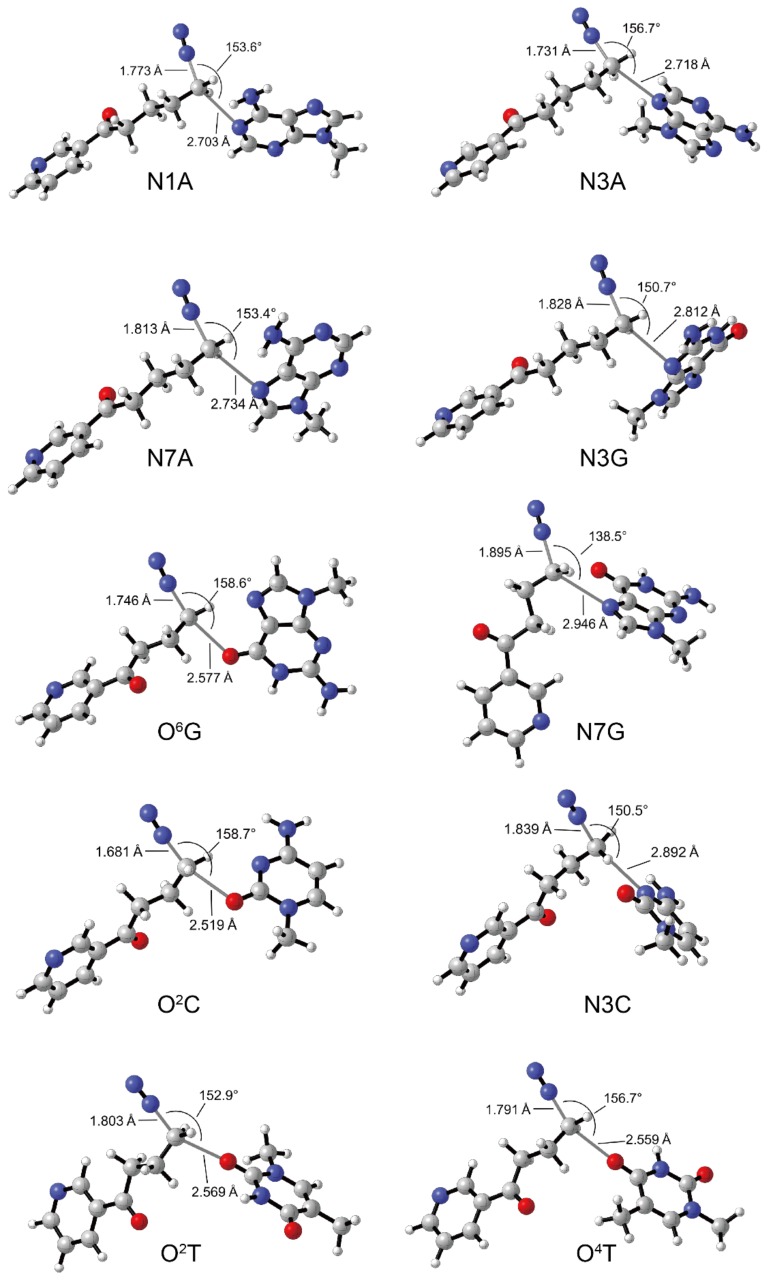
Optimized geometries and main parameters of the transition states for alkylation reactions with ten DNA base sites induced by the diazonium ions generated from 2′-hydroxylation of NNN. N1A = N1 position of adenine; N3A = N3 position of adenine; N7A = N7 position of adenine; N3G = N3 position of guanine; O^6^G = O^6^ position of guanine; N7G = N7 position of guanine; O^2^C = O^2^ position of cytosine; N3C = N3 position of cytosine; O^2^T = O^2^ position of thymine; O^4^T = O^4^ position of thymine.

The energy data obtained from alkylation at ten base sites induced by diazonium ions calculated at the B3LYP BSIV/BSIII theoretical level were listed in Table 1. The absolute energies (AE: a.u.) and relative energies (RE: kcal/mol) for the reaction species involved in alkylation reactions derived from 2′-hydroxylation of NNN with ten DNA base sites are shown in Table S4 in the Supplementary Materials. Transition state coordinate files were shown in the Supplementary Materials. All DNA alkylation products induced by 2′-hydroxylation products of NNN were POB-DNA adducts. We have verified the reliability of simplified N9-methylguanine model. The energy barriers obtained from the reactions of diazonium ions derived from 2′-hydroxylation of NNN with the nucleophilic O^6^ position of N9-methylguanine or 2′-deoxyguanosine at the B3LYP BSIV//BSIII level were similar (Table S5 in Supplementary Materials). The difference in the interatomic distances was below 0.008 Å. In theory, the diazonium ion generated from 2′-hydroxylation of NNN can attack various DNA base sites. In the present study, ten DNA base sites, including N1, N3, and N7 positions of adenine, N3, O^6^ and N7 positions of guanine, O^2^ and N3 positions of cytosine, and O^2^ and O^4^ positions of thymine, were selected to explore the DNA alkylation induced by the 2′-hydroxylation products of NNN. Among these base sites, N3 position of adenine, O^6^ and N7 positions of guanine have been identified as major reactive sites [64]. Alkylation reactions occurring at the above ten base sites are conducted via S_N_2 type reaction. We calculated all the ten proposed alkylation reactions and optimized the geometry structures. As shown in Figure 5, all transition states were similar in terms of the geometry structures, with the (N≡) N···C···N or (N≡) N···C···O angles ranging from 138.5° to 158.7°. The (N≡) N···C bond length was 1.681 to 1.895 Å; and the C···N or C···O bond length was 2.519 to 2.946 Å, in which the C···N bond was longer than C···O bond. Previous study indicated that the level of O^2^-POB dThd and 7-POB-Gua adducts were significantly higher than that of other POB-DNA adducts [10]. In this study, the energy barriers of most adduct formation reactions (0.86 to 4.72 kcal/mol) were below 5 kcal/mol, indicating these reactions occur easily. Therefore, it is unexpected that the calculation of the alkylation energy barriers on various DNA base sites cannot explain the site selectivity of DNA alkylation by the 2′-hydroxylation diazonium ion. 

It is well known that the metabolites of xenobiotics can lead to electrophilic intermediates that attack DNA, RNA, or proteins [65]. However, the electrophiles react discriminately with nucleophiles, and the degree of selectivity that occurs in electrophile–nucleophile interactions can be rationalized based on the theory of hard and soft acids and bases, preassociation mechanism, electrostatic interaction, and steric hindrance [66,67,68]. Thus, it may not be able to explain the experimental selectivity of alkylation reactions from the 2′-hydroxylation of NNN with quantum chemical calculations.

### 3.4. Docking Analysis

**Figure 6:**
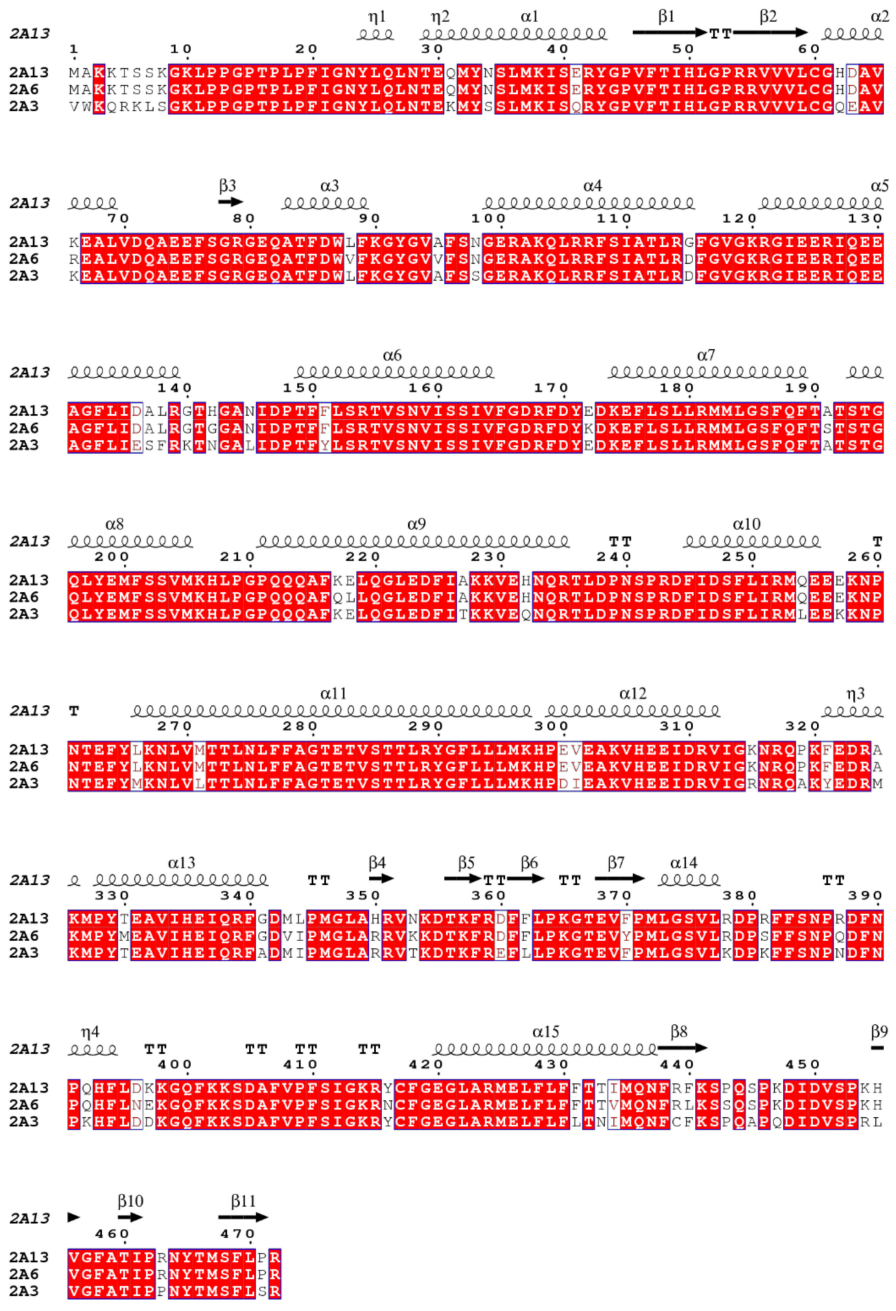
Sequence alignment of human 2A6, 2A13 and rat 2A3.

**Figure 7:**
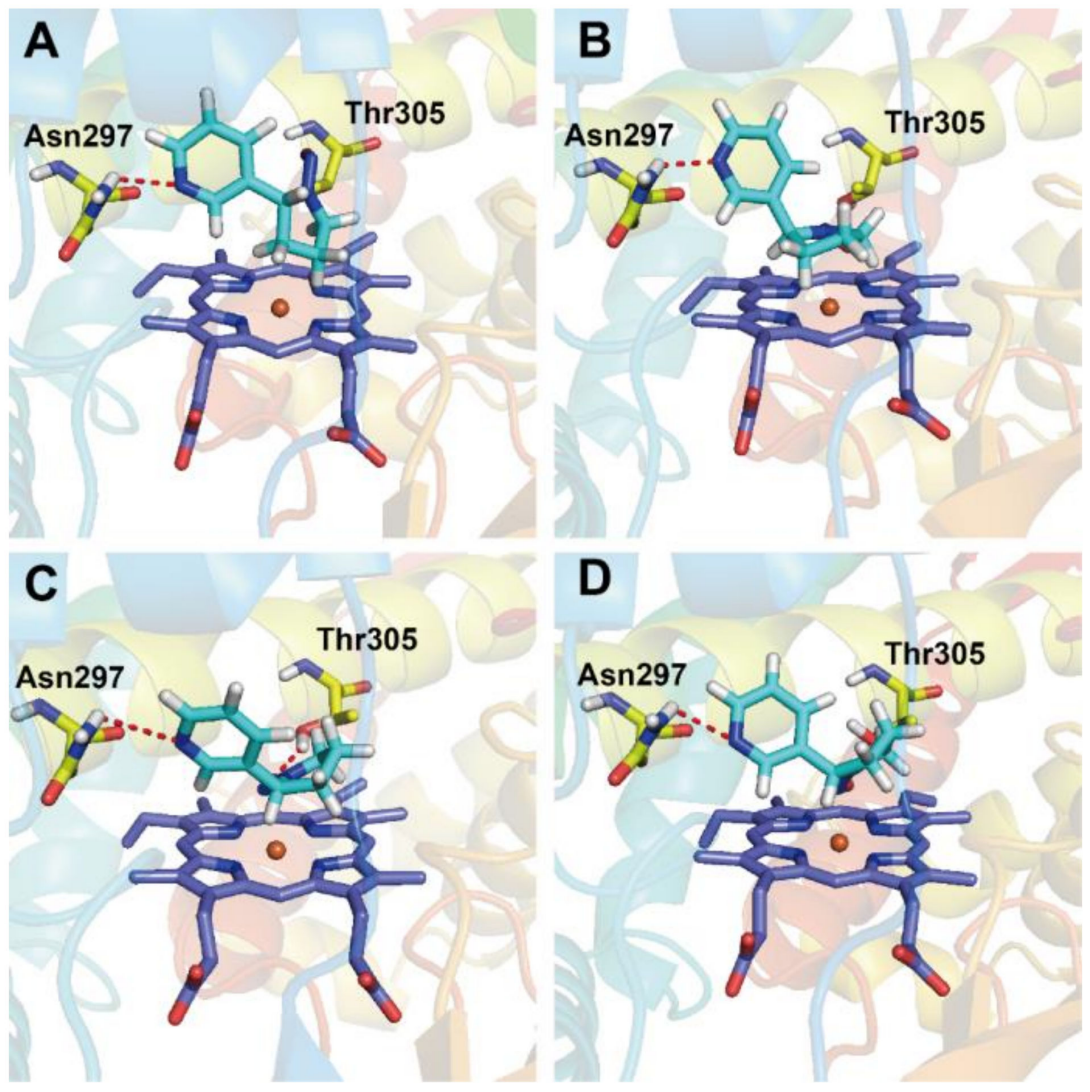
Optimal docking results of (*R*)- or (*S*)-NNN with different CYP450 enzymes. Docking conformations of (*R*)-NNN (**A**) and (*S*)-NNN (**B**) with rat CYP450 2A3 enzyme, and docking conformations of (*S*)-NNN with human CYP450 2A6 enzyme (**C**) and human CYP450 2A13 enzyme (**D**).

In order to better understand the carcinogenesis of NNN, we performed the docking study to investigate the interaction of CYP450s with NNN using GOLD 5.2 Suite software. Self-docking results indicated that the root mean square deviation (RMSD) of the ligand position between the docked pose with highest fitness score and the pose in protein–ligand crystal complex was 0.0517 Å (Figure S5 in the Supplementary Materials), which suggested that the docking conformation produced by GOLD was similar to the crystal structure. Thus, the GOLD program is suitable for performing the docking of nicotine analogues to CYP450 enzymes. The crystal structure of rat CYP450 2A3 was acquired from homology modeling using PDB entry 2P85 as the template. The crystal structures of all human CYP450s were downloaded from PDB database. (*R*)-NNN and (*S*)-NNN were docked with human CYP450 1A1, 2A6, 2A13, 2E1, 3A4, and rat CYP450 2A3. As shown in Figure 6, the modeled rat CYP450 2A3 has 89% similarity with human CYP450 2A13, 87% similarity with human CYP450 2A6, and 95% query coverage with human CYP450 2A13 and 2A6. The overlap of rat 2A3 and the human 2A13 protein crystal structures were shown in Figure S6 in the Supplementary Materials. The pose of each compound was selected based on the fitness score and the orientation. The docking scores were listed in Table 2. The compared docking conformations of rat 2A3 and human 2A6, 2A13 were shown in Figure 7. The detailed docking results were also displayed in Figure S7 in the Supplementary Materials.

Rat CYP450 2A3 is abundant in rat nose, which has been proven to be an efficient catalyst for the 2′-hydroxylation of (*S*)-NNN with kcat/K_M_ nearly twice as high as that of (*R*)-NNN [14]. Our docking results suggested that (*S*)-NNN presented an appropriate position at the active pocket of rat CYP450 2A3, which exhibited equally favored orientation for 2′- and 5′-position of (*S*)-NNN. By contrast, the conformation of (*R*)-NNN docked into rat CYP450 2A3 was favorable for 5′-hydroxylation but not for 2′-hydroxylation (Figure 7A,B). The docking score of (*R*)-NNN was lower than that of (*S*)-NNN, which also supported that (*S*)-NNN had better binding affinity with rat CYP450 2A3. (*S*)-NNN docked with human CYP450 2A6 and 2A13 had higher scores and better positions than with other enzymes, and presented a favorable position for 2′-hydroxylation (Figure 7C,D). The score of CYP450 2A6 docked with (*S*)-NNN was slightly higher than that of 2A13 and 1A1. Hydrogen bonds formed in CYP450 2A6 were between the nitrogen of the pyridine ring of (*S*)-NNN and Asn297 residue, and between the oxygen of the nitroso group of (*S*)-NNN and the Thr305 residue. For CYP450 2A13 and 1A1, only one hydrogen bond was formed between the nitrogen of the pyridine ring of (*S*)-NNN and Asn297 residue. Docking results of (*R*)-NNN and human CYP450s didn’t show favorable orientation to 2′- or 5′-hydroxylation, indicating the binding affinity of (*R*)-NNN with CYP450s was weak (Figure S7 in the Supplementary Materials). These observations suggested that (*S*)-NNN had better binding affinity with CYP450 2A6, 2A13, and 1A1 than (*R*)-NNN.

Several published papers have described the docking studies of tobacco chemicals with human CYP450 isoforms [69,70,71]. Docking studies using CYP450 2A13 predicted that NNK would be oriented in positions unfavorable for α-hydroxylation when the pyridine ring of NNK could not form hydrogen bond with Asn297 residue [69]. The authors suggested that the Asn297 residue was important for orienting NNK, (*S*)-NNN, and coumarin in the active center of CYP450 2A13, and mutation of this residue to Ala resulted in altered enzyme kinetics for these compounds [69]. Another study revealed the basis for the binding and access channel of nicotine and NNK in human CYP450 2A6 and 2A13 [50]. The binding of CYP450 2A enzyme with a variety of ligands appears to be largely sterically controlled by residue Asn297 as a key orienting feature [50]. The study by Xu et al. [70] indicated that the hydrogen bonds formed between NNK and CYP450 2A6 were different from those with 2A13. The hydrogen bonds in the NNK–2A6 complex formed between the oxygen in the carbonyl of NNK and Asn297 residue in CYP450 2A6 (3.0 Å). In the NNK–2A13 complex, the hydrogen bond formed between the pyridine nitrogen in NNK and Asn297 residue in CYP450 2A13 (3.2 Å). In this study, the hydrogen bond was mainly formed between the nitrogen of the pyridine ring in (*S*)-NNN and the Asn297 residue in CYP450s. Thus, residue Asn297 was also necessary for the metabolic activation of (*S*)-NNN, which was consistent with previous results [69]. It has been proven that CYP450s in extrahepatic tissues were important to chemical toxicity, because the toxicity of a given compound is tightly linked to its metabolic fate in the target tissue [72]. The binding forms of (*S*)-NNN with human CYP450 2A6, 2A13 and rat 2A3 provided rational explanations for the high levels of DNA adducts in rat nasal, oral, and esophageal tissue after exposure to (*S*)-NNN. Therefore, we suggest that the difference of metabolic activation and the organ specificity of NNN depends on the specific distribution of CYP450s and the affinity between chemicals and enzymes.

## 4. Conclusions

This research provided reasonable explanations for the metabolic activation and carcinogenesis of NNN. Quantum chemical calculations were applied to investigate the hydroxylation reactions of NNN, suggesting that the 2-(*S*)-pathway and 5-(*R*)-pathway were the prior metabolic pathways of NNN. Molecular docking study illustrated that (*S*)-NNN had higher affinity with the human CYP450 2A13, 2A6, 1A1 and rat 2A3. The hydrogen bonds were formed between the nitrogen of the pyridine ring in (*S*)-NNN and Asn297 residue in human CYP450 2A6 and 2A13 and formed between the nitrogen of the pyridine ring in (*R*/*S*)-NNN and the Asn297 residue in rat CYP450 2A3, which indicated that the Asn297 residue was a key orienting feature of steric control. The combined quantum chemical calculations and docking studies provided theoretical support for the experimental results, which may be used for further exploring the metabolic activation and carcinogenic effects of other TSNA and environmental carcinogens.

## Figures and Tables

**Table 1:** Free energy barriers of the transition states for alkylation reactions with ten DNA base sites induced by the 2′-hydroxylation diazonium ion ^a^.

Complex		Δ*G* ^‡^ (kcal/mol)								
	N1A	N3A	N7A	N3G	O^6^G	N7G	N3C	O^2^C	O^2^T	O^4^T
TS	2.42	0.92	3.85	0.86	2.35	4.72	5.03	1.71	3.82	2.79

^a^ Transition state energies were corrected with zero-point energy and free energy at 298.15 K with the B3LYP BSIV//BSIII level. N1A = N1 position of adenine; N3A = N3 position of adenine; N7A = N7 position of adenine; N3G = N3 position of guanine; O^6^G = O^6^ position of guanine; N7G = N7 position of guanine; O^2^C = O^2^ position of cytosine; N3C = N3 position of cytosine; O^2^T = O^2^ position of thymine; O^4^T = O^4^ position of thymine.

**Table 2:** Docking scores of (*R*)- or (*S*)-NNN with CYP450 enzymes.

CYP450 Enzymes	Scoring Function	
	(*R*)-NNN	(*S*)-NNN
Human 1A1-Chain A	55.29	70.67
Human 2A6-Chain A	63.88	73.98
Human 2A13-Chain A	58.32	73.55
Human 2E1-Chain A	53.79	62.59
Human 3A4	59.19	61.56
Rat 2A3	47.33	53.83

## Supplementary Materials

Supplementary materials can be found at http://www.mdpi.com/1660-4601/16/2/178/s1. Figure S1: Ramachandran Plot and statistics of modeled rat 2A3 enzymes, Figure S2: Optimized structures of 2-(R)-RC calculated by B3LYP and B3LYP-D3, Figure S3: Optimized geometries for 2′- and 5′-hydroxylation of (R)- and (S)-NNN catalyzed by Cpd I on low spin states. Bond lengths are given in angstroms, Figure S4: Optimized geometries for the decomposition of the hydroxylation products of (R)- and (S)-NNN. Bond lengths are given in angstroms, Figure S5: Self-docking structure of nicotine and CYP450 2A13, in which the brilliant blue nicotine is the native structure in the crystal complex and the pink nicotine is the redocked structure, Figure S6: Overlap of the rat 2A3 and the human 2A13 protein crystal structures, Figure S7: Detailed docking results of human CYP450 enzymes with NNN, Table S1: The absolute energies (AE: a.u.) and relative energies (RE: kcal/mol) for reaction species involved in 2′/5′-hydroxylation of (R)/(S)-NNN on doublet states, Table S2: The calculated spin densities for the reaction species involved in 2′/5′-hydroxylation of (R)/(S)-NNN on doublet states at UB3LYP-D3/B1 level, Table S3: The absolute energies (AE: a.u.) and relative energies (RE: kcal/mol) for reaction species involved in nonenzymatic decomposition of four α-hydroxyNNNs in the aqueous medium, Table S4: The absolute energies (AE: a.u.) and relative energies (RE: kcal/mol) for reaction species involved in alkylation of 2′-hydroxylation diazonium ion with ten DNA base site, Table S5: Comparison of free energies and optimized structures calculated for diazonium ions derived from 2′-hydroxylation of NNN binding with the nucleophilic O^6^ position of N9-methylguanine and 2′- deoxyguanosine at the BSV//BSIII level.
