# Supplementary material for: Metabolic Activation and Carcinogenesis of Tobacco-Specific Nitrosamine N’-Nitrosonornicotine (NNN): A Density Function Theory and Molecular Docking Study
Source: Int J Environ Res Public Health. 2019 Jan 9;16(2):178. doi: 10.3390/ijerph16020178 (PMC6352179; doi:10.3390/ijerph16020178)
Supplement: Supplementary file 1 [file ijerph-16-00178-s001.pdf]

## Supplementary Materials

**Table S1.** The absolute energies (AE: a.u.) and relative energies (RE: kcal/mol) for reaction species involved in 2′/5′-hydroxylation of (*R*)/(*S*)-NNN on doublet states.

| Species | BSI | BSII/BSI + ZPE + G | ZPE | G | BSII/BSI + ZPE + G |
|---|---|---|---|---|---|
| | AE | AE | AE | AE | RE |
| 2-R-RC | -2174.909905 | -2174.46881 | 0.485766 | 0.419933 | 1.80 |
| 2-R-TS1 | -2174.888941 | -2174.45315 | 0.479129 | 0.415421 | 11.62 |
| 2-R-IC | -2174.924462 | -2174.47308 | 0.482966 | 0.417769 | -10.70 |
| 2-R-PCOH | -2174.991339 | -2174.54369 | 0.487365 | 0.422796 | -45.19 |
| 2-S-RC | -2174.906852 | -2174.47168 | 0.485428 | 0.418655 | 0.00 |
| 2-S-TS1 | -2174.878811 | -2174.45822 | 0.478378 | 0.412522 | 8.44 |
| 2-S-IC | -2174.935672 | -2174.48954 | 0.483843 | 0.417873 | -11.21 |
| 2-S-PCOH | -2174.998594 | -2174.55263 | 0.488565 | 0.423542 | -50.80 |
| 5-R-RC | -2174.910152 | -2174.46832 | 0.485884 | 0.420006 | 2.11 |
| 5-R-TS1 | -2174.873639 | -2174.45558 | 0.478575 | 0.413441 | 10.10 |
| 5-R-IC | -2174.932561 | -2174.47925 | 0.481973 | 0.416366 | -4.75 |
| 5-R-PCOH | -2175.000425 | -2174.55196 | 0.489439 | 0.425538 | -50.38 |
| 5-S-RC | -2174.903567 | -2174.47441 | 0.485548 | 0.41808 | -3.82 |
| 5-S-TS1 | -2174.873772 | -2174.45977 | 0.47875 | 0.412345 | 5.37 |
| 5-S-IC | -2174.926746 | -2174.47849 | 0.481967 | 0.416072 | -6.38 |
| 5-S-PCOH | -2174.988325 | -2174.54667 | 0.48814 | 0.423397 | -49.17 |
| 2-R-$^4$RC | -2174.871610 | -2174.468318 | 0.486614 | 0.419885 | 2.42 |
| 2-R-$^4$TS1 | -2174.886438 | -2174.455538 | 0.478547 | 0.413413 | 10.44 |
| 2-S-$^4$RC | -2174.906848 | -2174.472181 | 0.485458 | 0.418115 | 0.00 |
| 2-S-$^4$TS1 | -2174.876754 | -2174.458697 | 0.478092 | 0.412206 | 8.46 |
| 5-R-$^4$RC | -2174.909909 | -2174.468745 | 0.485898 | 0.419352 | -0.27 |
| 5-R-$^4$TS1 | -2174.874891 | -2174.455807 | 0.479092 | 0.413018 | 7.85 |
| 5-S-$^4$RC | -2174.903468 | -2174.474703 | 0.485571 | 0.417574 | -1.58 |
| 5-S-$^4$TS1 | -2174.868000 | -2174.458214 | 0.480343 | 0.414295 | 8.77 |

ZPE: Zero-point energy; G: Gibbs free energy correction; RC = reactant complex in low-spin state, TS1 = transition state of H-abstraction in low-spin state, IC = intermediate complex with H abstracted by Cpd I in low-spin state, PCOH = hydroxylation product +Fe (Por SH) in low-spin state; $^4$RC=reactant complex in high-spin state; $^4$TS1=transition state of H-abstraction in high-spin state.

**Table S2.** The calculated spin densities for the reaction species involved in 2′/5′-hydroxylation of (*R*)/(*S*)-NNN on doublet states at UB3LYP-D3/B1 level.

| Species | Cpd I | | | | Substrate | | | |
|---|---|---|---|---|---|---|---|---|
| | Fe | O | Por | SH | H | C | N | Rest |
| 2-R-RC | 1.25 | 0.83 | -0.54 | -0.54 | 0.00 | 0.00 | 0.00 | 0.00 |
| 2-R-TS1 | 1.07 | 0.58 | -0.55 | -0.42 | -0.06 | 0.27 | -0.03 | 0.14 |
| 2-R-IC | 1.31 | 0.07 | -0.27 | -0.06 | -0.04 | 0.01 | 0.00 | -0.02 |
| 2-R-PC | 1.11 | 0.00 | -0.10 | -0.01 | 0.00 | 0.00 | 0.00 | 0.00 |
| 2-S-RC | 1.25 | 0.83 | -0.54 | -0.54 | 0.00 | 0.00 | 0.00 | 0.00 |
| 2-S-TS1 | 1.04 | 0.57 | -0.54 | -0.44 | -0.06 | 0.31 | -0.01 | 0.13 |
| 2-S-IC | 1.45 | 0.02 | -0.44 | -0.06 | -0.01 | 0.00 | -0.01 | 0.05 |
| 2-S-PC | 1.09 | 0.00 | -0.08 | 0.00 | 0.00 | 0.00 | 0.00 | 0.00 |
| 5-R-RC | 1.25 | 0.83 | -0.52 | -0.56 | 0.00 | 0.00 | 0.00 | 0.00 |
| 5-R-TS1 | 1.06 | 0.54 | -0.52 | -0.38 | -0.05 | 0.28 | -0.03 | 0.11 |
| 5-R-IC | 1.21 | 0.07 | -0.21 | -0.04 | -0.01 | 0.00 | 0.00 | -0.01 |
| 5-R-PC | 1.09 | 0.00 | -0.09 | 0.00 | 0.00 | 0.00 | 0.00 | 0.00 |
| 5-S-RC | 1.23 | 0.85 | -0.53 | -0.56 | 0.00 | 0.00 | 0.00 | 0.00 |
| 5-S-TS1 | 1.35 | 0.36 | -0.44 | -0.23 | -0.03 | 0.02 | 0.05 | 0.03 |
| 5-S-IC | 1.16 | 0.07 | -0.18 | -0.03 | -0.02 | 0.00 | 0.00 | 0.00 |
| 5-S-PC | 1.10 | 0.00 | -0.09 | -0.01 | 0.00 | 0.00 | 0.00 | 0.00 |

| | | | | | | | |
|---|---|---|---|---|---|---|---|---|
| 2-R-$^4$RC | 1.14 | 0.88 | 0.50 | 0.47 | 0.00 | 0.00 | 0.00 | 0.00 |
| 2-R-$^4$TS1 | 0.96 | 0.66 | 0.55 | 0.40 | -0.06 | 0.34 | -0.02 | 0.17 |
| 2-S-$^4$RC | 1.14 | 0.88 | 0.48 | 0.49 | 0.00 | 0.00 | 0.00 | 0.00 |
| 2-S-$^4$TS1 | 0.94 | 0.66 | 0.53 | 0.44 | -0.06 | 0.36 | 0.01 | 0.15 |
| 5-R-$^4$RC | 1.14 | 0.88 | 0.45 | 0.52 | 0.00 | 0.00 | 0.00 | 0.00 |
| 5-R-$^4$TS1 | 1.21 | 0.66 | 0.25 | 0.41 | -0.04 | 0.35 | 0.00 | 0.14 |
| 5-S-$^4$RC | 1.12 | 0.89 | 0.46 | 0.51 | 0.00 | 0.00 | 0.00 | 0.00 |
| 5-S-$^4$TS1 | 1.36 | 0.70 | 0.01 | 0.45 | -0.03 | 0.32 | 0.03 | 0.14 |

**Table S3.** The absolute energies (AE: a.u.) and relative energies (RE: kcal/mol) for reaction species involved in nonenzymatic decomposition of four $\alpha$-hydroxyNNNs in the aqueous medium.

| Species | BSIII | BSIV/BSIII+ZPE+G | ZPE | G | BSII/BSI+ZPE+G |
|---|---|---|---|---|---|
| | AE | AE | AE | AE | RE |
| 2-R-PC$_{OH}$' | -664.2542336 | -664.2575942 | 0.199242 | 0.160076 | -0.02 |
| 2-R-TS2 | -664.2221708 | -664.2186251 | 0.196511 | 0.158565 | 18.02 |
| 2-R-PC | -664.2603075 | -664.2476555 | 0.197222 | 0.154613 | -8.96 |
| 2-S-PC$_{OH}$' | -664.2544469 | -664.0785881 | 0.199647 | 0.160924 | 0.00 |
| 2-S-TS2 | -664.2225143 | -664.0498716 | 0.19651 | 0.158562 | 18.02 |
| 2-S-PC | -664.2603075 | -664.0864213 | 0.197334 | 0.155391 | -4.92 |
| 5-R-PC$_{OH}$' | -664.2589800 | -664.0796655 | 0.200435 | 0.161567 | -0.68 |
| 5-R-TS2 | -664.2193652 | -664.0504504 | 0.196928 | 0.158307 | 17.65 |
| 5-R-PC | -664.2453873 | -664.0781517 | 0.196533 | 0.154627 | 0.27 |
| 5-S-PC$_{OH}$' | -664.2575942 | -664.0782284 | 0.200454 | 0.16148 | 0.23 |
| 5-S-TS2 | -664.2186251 | -664.0471018 | 0.196504 | 0.157764 | 19.76 |
| 5-S-PC | -664.2476555 | -664.0821310 | 0.196589 | 0.153709 | -2.22 |

ZPE: Zero-point energy; G: Gibbs free energy correction; PCOH' = hydroxylation product, TS2 = transition state of decomposition, PC = decomposition products.

**Table S4.** The absolute energies (AE: a.u.) and relative energies (RE: kcal/mol) for reaction species involved in alkylation of 2′-hydroxylation diazonium ion with ten DNA base site.

| Species | BSIII | BSIV/BSIII+ZPE+G | ZPE | G | BSII/BSI+ZPE+G |
|---|---|---|---|---|---|
| | AE | AE | AE | AE | RE |
| N1A-R | -1134.179980 | -1133.925416 | 0.351251 | 0.289941 | 0 |
| N1A-TS | -1134.175372 | -1133.921559 | 0.349479 | 0.288645 | 2.42 |
| N1A-P | -1134.259980 | -1134.017051 | 0.347382 | 0.292816 | -57.50 |
| N3A-R | -1134.182703 | -1133.927191 | 0.350565 | 0.291143 | 0 |
| N3A-TS | -1134.172331 | -1133.925721 | 0.348854 | 0.287419 | 0.92 |
| N3A-P | -1134.271204 | -1134.015472 | 0.346092 | 0.293083 | -55.40 |
| N7A-R | -1134.181048 | -1133.926850 | 0.351073 | 0.289243 | 0 |
| N7A-TS | -1134.173762 | -1133.920706 | 0.349250 | 0.288492 | 3.85 |
| N7A-P | -1134.268722 | -1134.020627 | 0.345028 | 0.290729 | -58.85 |
| N3G-R | -1209.411945 | -1209.192458 | 0.35519 | 0.292878 | 0 |
| N3G-TS | -1209.398974 | -1209.191091 | 0.352842 | 0.289695 | 0.86 |
| N3G-P | -1209.485015 | -1209.270923 | 0.355568 | 0.289030 | -49.24 |
| O$^6$G-R | -1170.131922 | -1169.931381 | 0.327037 | 0.267783 | 0 |
| O$^6$G-TS | -1170.123654 | -1169.927631 | 0.325129 | 0.265411 | 2.35 |
| O$^6$G-P | -1170.223654 | -1170.01579 | 0.308123 | 0.254258 | -52.97 |
| N7G-R | -1170.131368 | -1169.931997 | 0.326962 | 0.267178 | 0 |
| N7G-TS | -1170.120256 | -1169.924483 | 0.324704 | 0.263876 | 4.72 |
| N7G-P | -1170.20868 | -1170.025414 | 0.321295 | 0.267564 | -58.62 |
| N3C-R | -1022.496133 | -1022.291101 | 0.308500 | 0.250217 | 0 |
| N3C-TS | -1022.487422 | -1022.283081 | 0.307300 | 0.250117 | 5.03 |
| N3C-P | -1022.572632 | -1022.377067 | 0.304397 | 0.254682 | -53.94 |
| O$^2$C-R | -1022.491368 | -1022.289422 | 0.308531 | 0.251184 | 0 |
| O$^2$C-TS | -1022.489631 | -1022.286689 | 0.307511 | 0.250255 | 1.71 |
| O$^2$C-P | -1022.568067 | -1022.365364 | 0.304167 | 0.253511 | -47.65 |

| | | | | | |
|---|---|---|---|---|---|
| $O^2T$-R | -1081.680433 | -1081.477794 | 0.324679 | 0.262497 | 0 |
| $O^2T$-TS | -1081.672038 | -1081.471710 | 0.322451 | 0.261252 | 3.82 |
| $O^2T$-P | -1081.762038 | -1081.572970 | 0.315834 | 0.262043 | -59.72 |
| $O^4T$-R | -1081.685124 | -1081.476827 | 0.324951 | 0.263820 | 0 |
| $O^4T$-TS | -1081.676775 | -1081.472373 | 0.322565 | 0.260928 | 2.79 |
| $O^4T$-P | -1081.754737 | -1081.547165 | 0.314665 | 0.259372 | -44.14 |

N1A = N1 position of adenine, N3A = N3 position of adenine, N7A = N7 position of adenine, N3G = N3 position of guanine, $O^6G$ = $O^6$ position of guanine, N7G = N7 position of guanine, $O^2C$ = $O^2$ position of cytosine, N3C = N3 position of cytosine, $O^2T$ = $O^2$ position of thymine, $O^4T$ = $O^4$ position of thymine.; R = reactant complex, TS = transition state of alkylation, P = alkylation products.

**Table S5**. Comparison of free energies and optimized structures calculated for diazonium ions derived from 2'-hydroxylation of NNN binding with the nucleophilic $O^6$ position of N9-methylguanine and 2'- deoxyguanosine at the BSV//BSIII level.

| Complex | $\Delta G^{\ddagger}$ | Frequency ($i$ cm$^{-1}$) | C-N distance (Å) | C-O distance (Å) |
|---|---|---|---|---|
| RC(N9-methylguanine) | 0.00 | - | - | - |
| TS(N9-methylguanine) | 2.35 | -355.23 | 1.746 | 2.577 |
| RC(2'-deoxyguanosine) | 0.00 | | | |
| TS(2'-deoxyguanosine) | 1.68 | -342.84 | 1.738 | 2.570 |

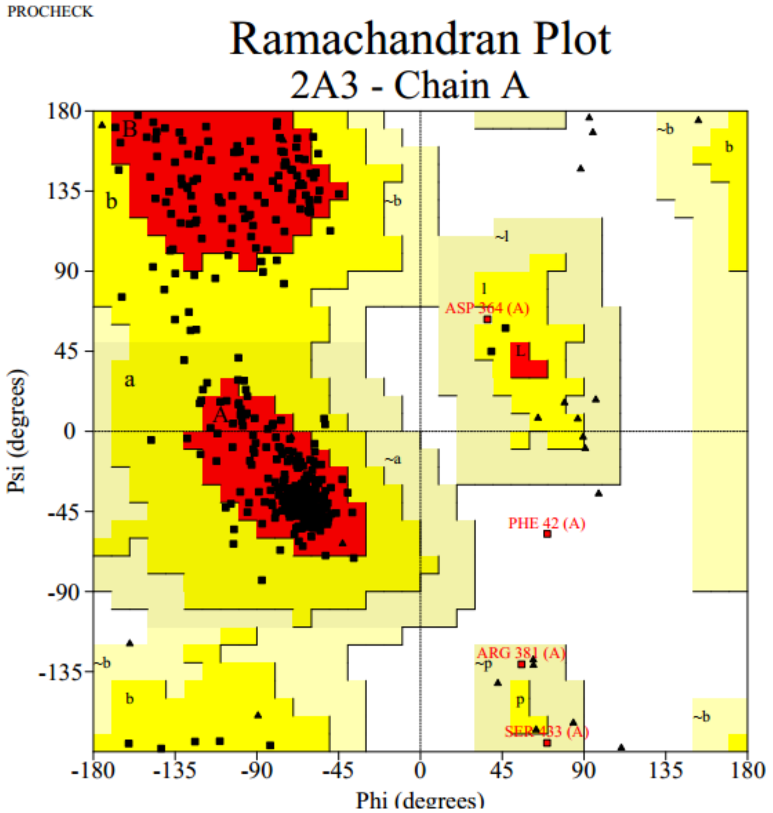


**Figure S1.** Ramachandran Plot and statistics of modeled rat 2A3 enzymes.

**Plot statistics**

Residues in most favoured regions [A,B,L] 353 **88.3%**

Residues in additional allowed regions [a,b,l,p] 43 10.8%

Residues in generously allowed regions [~a,~b,~l,~p] 3 0.8%

Residues in disallowed regions 1 0.3%

---- ---------- ------

**SAVES validation**

Vertify3D **98%** of residues had an averaged 3D-1D score ≥0.2.

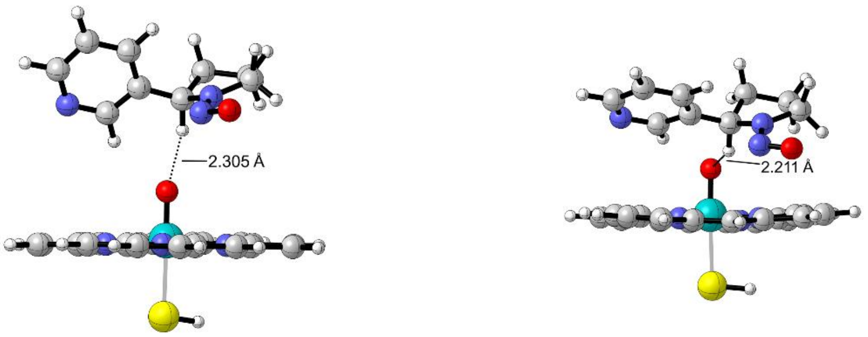


**Figure S2.** Optimized structures of 2-(*R*)-RC calculated by B3LYP and B3LYP-D3.

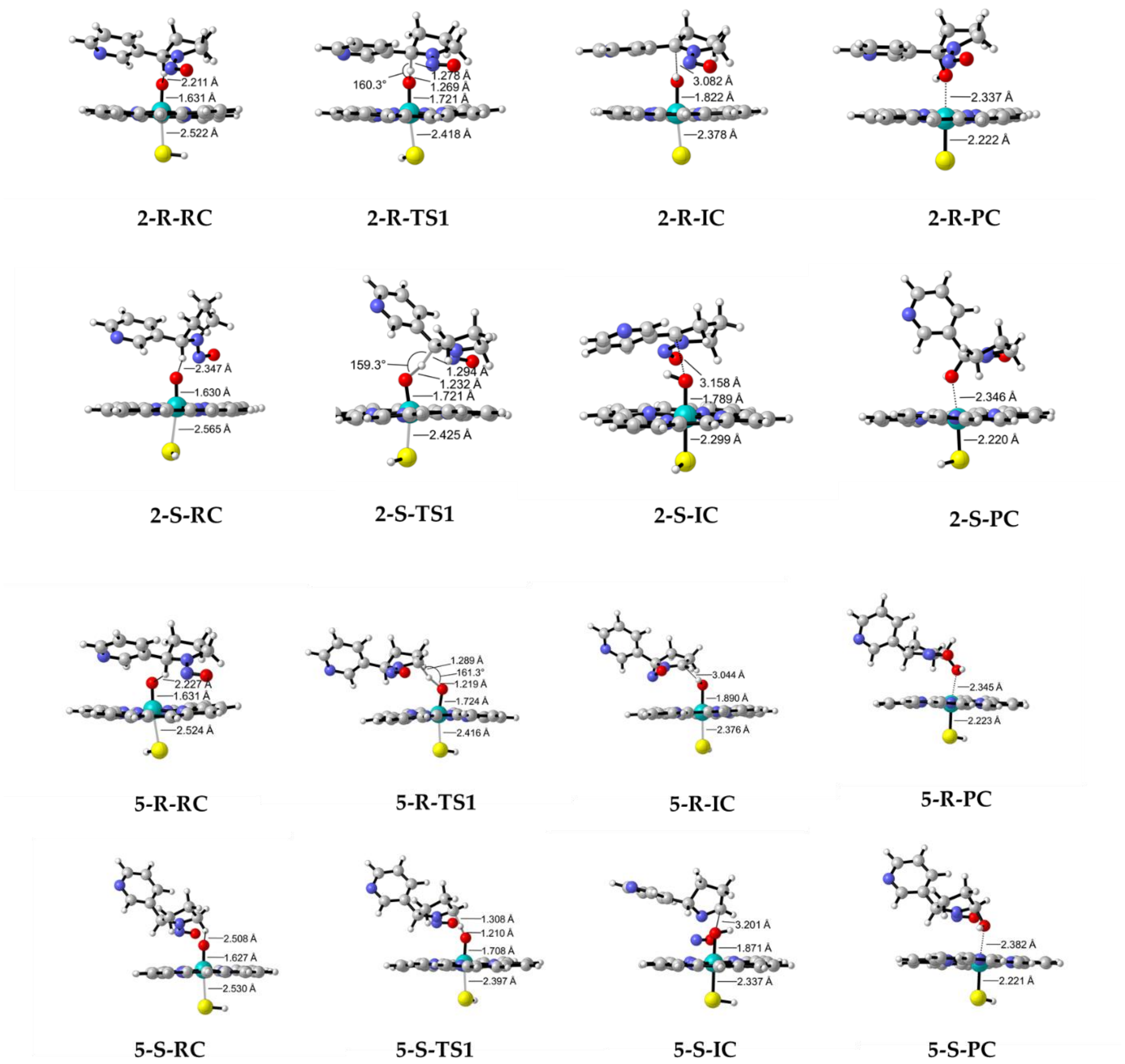


**Figure S3.** Optimized geometries for 2'- and 5'-hydroxylation of *(R)*- and *(S)*-NNN catalyzed by Cpd I on low spin states. Bond lengths are given in angstroms.

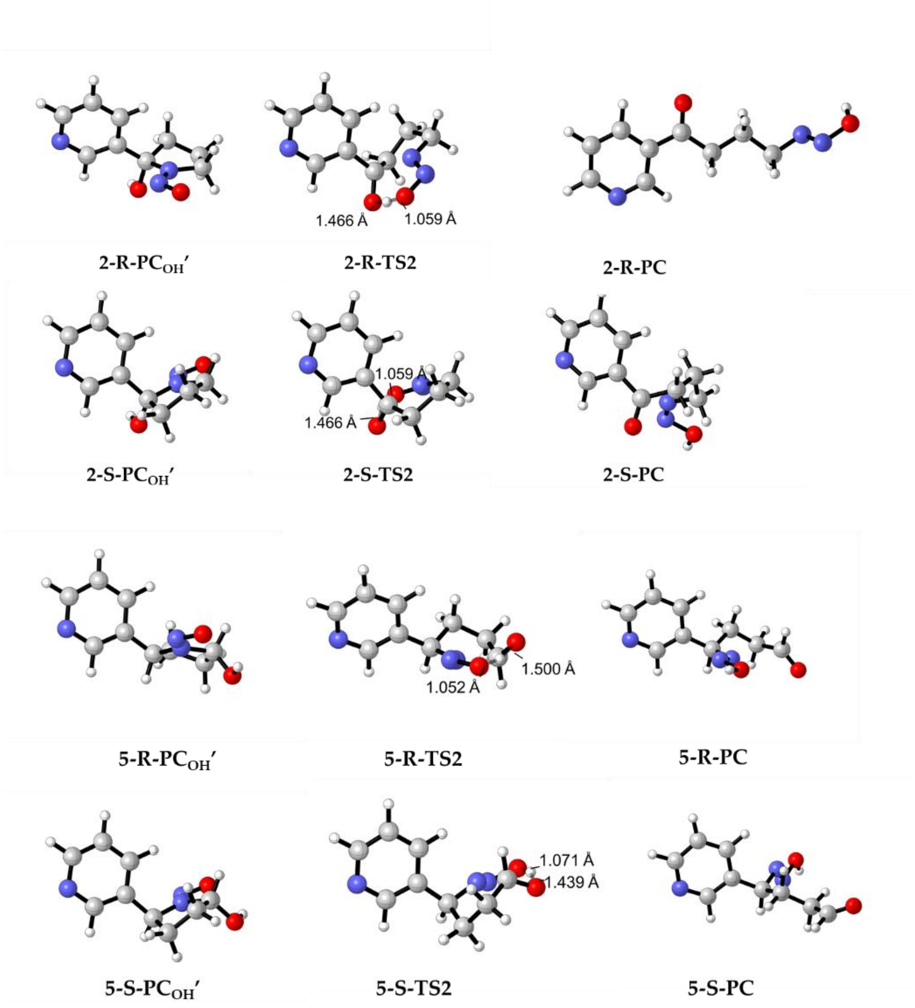


**Figure S4.** Optimized geometries for the decomposition of the hydroxylation products of *(R)*- and *(S)*-NNN. Bond lengths are given in angstroms.

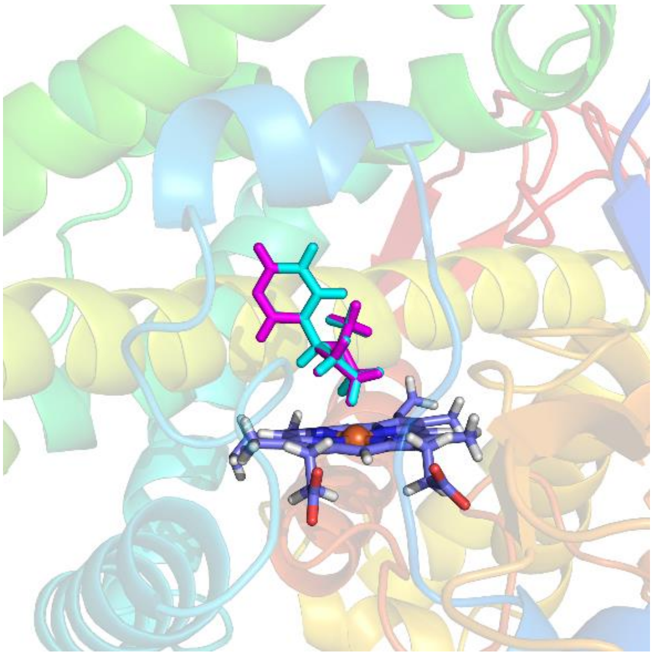

**Figure S5.** Self-docking structure of nicotine and CYP450 2A13, in which the brilliant blue nicotine is the native structure in the crystal complex and the pink nicotine is the redocked structure.

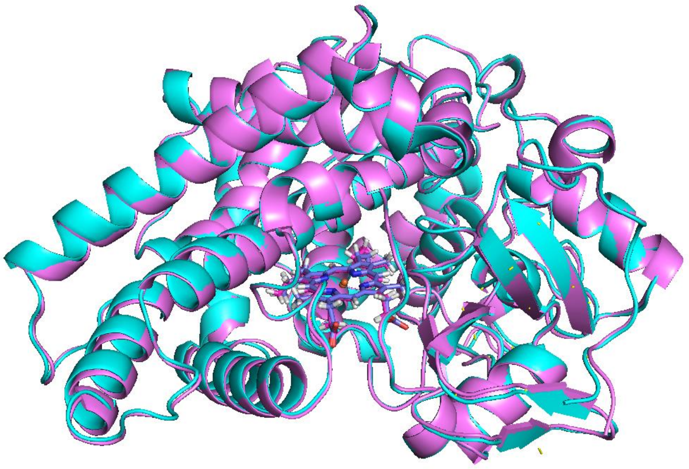

**Figure S6.** Overlap of the rat 2A3 and the human 2A13 protein crystal structures.

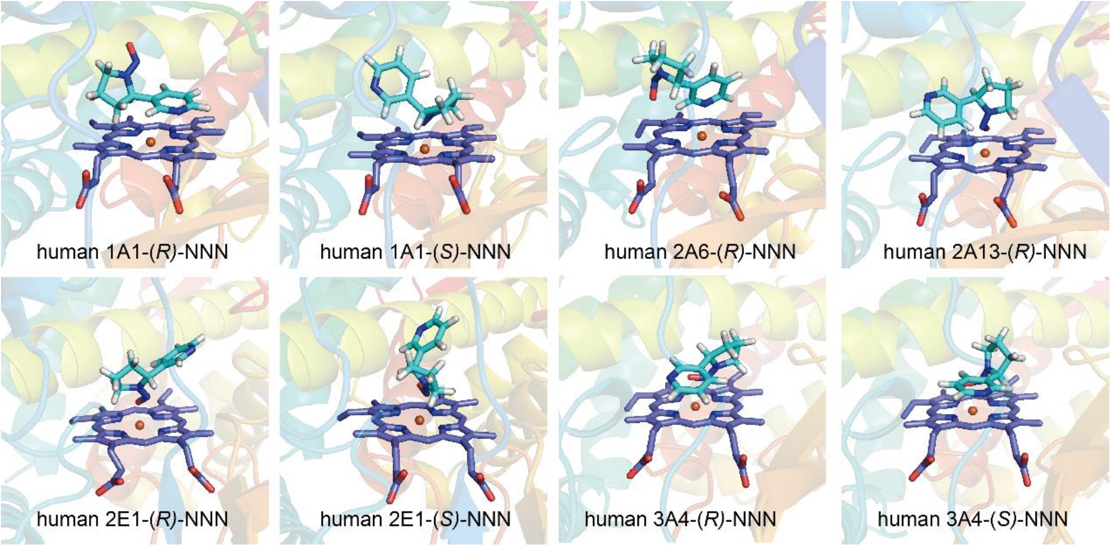


**Figure S7.** Detailed docking results of human CYP450 enzymes with NNN.

**Cartesian coordinates of TS structures discussed in this work.**

**2-R-TS1**

| | | | |
|---|---|---|---|
| Fe | -1.10898 | 0.19663 | 0.42756 |
| O | -0.35248 | 0.04937 | -1.11102 |
| C | 0.12539 | -2.32299 | 1.61681 |
| C | -0.0072 | -3.75876 | 1.61536 |
| C | -1.09748 | -4.0487 | 0.8512 |
| C | -1.63765 | -2.78881 | 0.39733 |
| N | -0.87196 | -1.75043 | 0.86896 |
| H | 0.67041 | -4.43618 | 2.116 |
| H | -1.51576 | -5.01779 | 0.61341 |
| C | 1.1468 | 1.84659 | 1.57397 |
| C | 2.36645 | 1.7452 | 2.33953 |
| C | 2.49777 | 0.4378 | 2.69308 |
| C | 1.36662 | -0.25743 | 2.12941 |
| N | 0.5608 | 0.61382 | 1.44391 |
| H | 3.02129 | 2.57632 | 2.56266 |
| H | 3.28505 | -0.03502 | 3.26399 |
| C | -2.49974 | 2.73521 | -0.53589 |
| C | -2.30517 | 4.16141 | -0.64435 |
| C | -1.10171 | 4.43578 | -0.06889 |
| C | -0.57418 | 3.17872 | 0.40362 |
| N | -1.44105 | 2.15895 | 0.11425 |
| H | -3.00105 | 4.84395 | -1.11374 |
| H | -0.60296 | 5.38992 | 0.03505 |
| C | -3.37295 | -1.46596 | -0.76473 |
| C | -4.61462 | -1.35972 | -1.49667 |
| C | -4.85924 | -0.03202 | -1.66479 |
| C | -3.7586 | 0.67398 | -1.05052 |
| N | -2.87367 | -0.21769 | -0.49552 |
| H | -5.2096 | -2.20217 | -1.82356 |
| H | -5.6937 | 0.4431 | -2.16322 |
| C | -2.78335 | -2.66144 | -0.37784 |
| C | -3.59412 | 2.05063 | -1.05712 |
| C | 0.63599 | 3.03597 | 1.07179 |
| C | 1.15568 | -1.62626 | 2.23233 |
| H | -4.3649 | 2.64244 | -1.54083 |
| H | 1.23009 | 3.93126 | 1.21889 |
| H | 1.89511 | -2.20706 | 2.77152 |
| H | -3.28408 | -3.57868 | -0.67179 |
| S | -2.52894 | 0.19803 | 2.38515 |
| H | -3.35595 | 1.18033 | 1.97326 |
| C | 2.34058 | 1.77718 | -1.51278 |

| | | | |
|---|---|---|---|
| C | 2.878 | 0.50493 | -1.23085 |
| C | 4.179 | 0.45927 | -0.71509 |
| C | 4.87073 | 1.65463 | -0.52856 |
| C | 4.24372 | 2.85669 | -0.85381 |
| N | 2.99478 | 2.9278 | -1.33633 |
| H | 4.63274 | -0.48739 | -0.45205 |
| H | 1.31415 | 1.85153 | -1.86472 |
| H | 5.88165 | 1.65275 | -0.13262 |
| H | 4.76117 | 3.80484 | -0.71696 |
| C | 2.01539 | -0.67572 | -1.51743 |
| C | 1.65579 | -0.98339 | -2.97804 |
| H | 0.85299 | -0.3481 | -1.09985 |
| C | 0.94817 | -2.34368 | -2.85613 |
| H | 2.57474 | -1.05568 | -3.57546 |
| H | 1.02021 | -0.20501 | -3.40228 |
| C | 1.73432 | -3.07382 | -1.75608 |
| H | -0.08005 | -2.17536 | -2.52468 |
| H | 0.93115 | -2.90627 | -3.79243 |
| H | 1.12113 | -3.68045 | -1.08569 |
| H | 2.54166 | -3.70909 | -2.13625 |
| N | 2.34202 | -1.96805 | -0.99032 |
| N | 3.05906 | -2.17736 | 0.10941 |
| O | 3.17185 | -3.37364 | 0.40759 |

**2-S-TS1**

| | | | |
|---|---|---|---|
| Fe | 1.24448 | -0.2065 | 0.31905 |
| O | 0.07266 | 0.57348 | -0.67029 |
| C | 1.859 | 2.59233 | 1.34783 |
| C | 2.65766 | 3.7671 | 1.0779 |
| C | 3.44362 | 3.47142 | 0.00854 |
| C | 3.11502 | 2.12119 | -0.38792 |
| N | 2.16048 | 1.59964 | 0.45377 |
| H | 2.60952 | 4.68629 | 1.64637 |
| H | 4.17205 | 4.09826 | -0.48826 |
| C | -0.55672 | -0.70672 | 2.72229 |
| C | -1.13899 | -0.03693 | 3.85945 |
| C | -0.66979 | 1.23893 | 3.84162 |
| C | 0.21238 | 1.34544 | 2.70249 |
| N | 0.26064 | 0.15007 | 2.02895 |
| H | -1.83758 | -0.49408 | 4.54622 |
| H | -0.88583 | 2.04728 | 4.52724 |
| C | 0.65903 | -2.99832 | -0.70058 |
| C | -0.05936 | -4.20744 | -0.37871 |
| C | -0.68083 | -3.98628 | 0.8145 |

| | | | |
|---|---|---|---|
| C | -0.35617 | -2.63601 | 1.20485 |
| N | 0.45325 | -2.0486 | 0.26755 |
| H | -0.05945 | -5.10419 | -0.98405 |
| H | -1.30215 | -4.65982 | 1.38962 |
| C | 3.28068 | 0.19188 | -1.9198 |
| C | 3.79429 | -0.4371 | -3.1133 |
| C | 3.16744 | -1.64147 | -3.21988 |
| C | 2.28233 | -1.74753 | -2.08478 |
| N | 2.37126 | -0.62356 | -1.30501 |
| H | 4.5305 | 0.00238 | -3.7731 |
| H | 3.28519 | -2.39935 | -3.98299 |
| C | 3.64844 | 1.46352 | -1.48305 |
| C | 1.48118 | -2.84881 | -1.81263 |
| C | -0.82578 | -2.01609 | 2.35711 |
| C | 0.93263 | 2.48625 | 2.37598 |
| H | 1.52925 | -3.68096 | -2.50816 |
| H | -1.50148 | -2.58037 | 2.98993 |
| H | 0.78703 | 3.35919 | 3.00419 |
| H | 4.39015 | 1.99453 | -2.07134 |
| S | 3.05238 | -0.82268 | 1.81315 |
| H | 4.05579 | -0.50667 | 0.96937 |
| C | -4.03154 | 1.87353 | -0.77188 |
| C | -2.84184 | 1.35942 | -1.30139 |
| C | -2.09791 | 2.1994 | -2.1545 |
| C | -3.62155 | 3.89574 | -1.9984 |
| H | -1.14659 | 1.84751 | -2.54694 |
| H | -4.63607 | 1.27988 | -0.09569 |
| H | -3.90697 | 4.90279 | -2.29747 |
| C | -2.12187 | -0.9712 | -2.19863 |
| C | -3.29537 | -1.96318 | -2.07158 |
| H | -1.16524 | -1.49003 | -2.08737 |
| H | -2.10749 | -0.4394 | -3.15164 |
| C | -3.49007 | -2.10948 | -0.55779 |
| H | -4.19818 | -1.5381 | -2.52303 |
| H | -3.09258 | -2.92242 | -2.55452 |
| H | -4.5202 | -2.28217 | -0.238 |
| H | -2.86306 | -2.89372 | -0.12286 |
| N | -3.0456 | -0.79726 | -0.05629 |
| N | -3.24656 | -0.38017 | 1.19015 |
| O | -3.85459 | -1.19042 | 1.89754 |
| C | -2.31805 | -0.0091 | -1.014 |
| H | -1.10875 | 0.2277 | -0.62039 |
| N | -2.46959 | 3.43196 | -2.50554 |
| C | -4.42855 | 3.16004 | -1.13061 |

| | | | |
|---|---|---|---|
| H | -5.34998 | 3.5852 | -0.7446 |

**5-R-TS1**

| | | | |
|---|---|---|---|
| Fe | 1.564 | -0.03725 | 0.26818 |
| O | 1.01823 | -0.05628 | -1.36655 |
| C | 3.48359 | 2.11592 | -0.68998 |
| C | 4.77209 | 2.3159 | -1.31547 |
| C | 5.32725 | 1.08462 | -1.4728 |
| C | 4.37146 | 0.12917 | -0.95975 |
| N | 3.26603 | 0.78208 | -0.47172 |
| H | 5.18136 | 3.28049 | -1.58474 |
| H | 6.28545 | 0.82554 | -1.90319 |
| C | -0.27205 | 2.09578 | 1.43458 |
| C | -0.43033 | 3.52734 | 1.51738 |
| C | 0.60494 | 4.07461 | 0.82883 |
| C | 1.40956 | 2.97803 | 0.34152 |
| N | 0.85215 | 1.7797 | 0.71116 |
| H | -1.24742 | 4.02548 | 2.02065 |
| H | 0.82534 | 5.11975 | 0.65859 |
| C | -0.30827 | -2.19745 | 1.27334 |
| C | -1.5005 | -2.40167 | 2.05977 |
| C | -1.93843 | -1.16486 | 2.4346 |
| C | -1.02507 | -0.21073 | 1.85521 |
| N | -0.04406 | -0.85386 | 1.14856 |
| H | -1.91957 | -3.36853 | 2.30521 |
| H | -2.79637 | -0.90534 | 3.04015 |
| C | 3.57653 | -2.17747 | -0.58493 |
| C | 3.72391 | -3.60749 | -0.71879 |
| C | 2.57473 | -4.16368 | -0.24479 |
| C | 1.7365 | -3.07098 | 0.18913 |
| N | 2.36611 | -1.8744 | -0.02354 |
| H | 4.59467 | -4.1015 | -1.129 |
| H | 2.3076 | -5.21008 | -0.17891 |
| C | 4.52653 | -1.24688 | -0.99874 |
| C | 0.48914 | -3.22448 | 0.78414 |
| C | -1.14412 | 1.17103 | 1.98322 |
| C | 2.60954 | 3.13562 | -0.33825 |
| H | 0.13177 | -4.23943 | 0.92903 |
| H | -1.99068 | 1.55047 | 2.54518 |
| H | 2.91518 | 4.15109 | -0.56958 |
| H | 5.44946 | -1.63315 | -1.41977 |
| S | 2.75776 | 0.20264 | 2.3547 |
| H | 3.7769 | -0.62212 | 2.03988 |
| C | -5.11744 | -0.93038 | 0.59143 |

| | | | |
|---|---|---|---|
| C | -4.54325 | -0.32742 | -0.5321 |
| C | -5.35471 | 0.51441 | -1.30046 |
| C | -6.67699 | 0.70934 | -0.91535 |
| C | -7.14626 | 0.05113 | 0.22544 |
| N | -6.38902 | -0.7585 | 0.97395 |
| H | -4.9491 | 1.0176 | -2.17309 |
| H | -4.51172 | -1.58682 | 1.21422 |
| H | -7.33484 | 1.36091 | -1.48189 |
| H | -8.17608 | 0.18459 | 0.55217 |
| C | -3.12094 | -0.65129 | -0.91015 |
| C | -2.96859 | -1.55531 | -2.15222 |
| H | -2.62204 | -1.1233 | -0.05867 |
| C | -1.53833 | -1.2564 | -2.64858 |
| H | -3.70985 | -1.25771 | -2.90229 |
| H | -3.13739 | -2.60657 | -1.90684 |
| C | -1.28656 | 0.19114 | -2.23292 |
| H | -0.81051 | -1.90232 | -2.14493 |
| H | -1.42142 | -1.40461 | -3.72496 |
| H | -0.14053 | 0.20303 | -1.64406 |
| H | -1.12775 | 0.96224 | -2.98726 |
| N | -2.31909 | 0.51831 | -1.32267 |
| N | -2.52907 | 1.73548 | -0.80171 |
| O | -1.7414 | 2.58871 | -1.21615 |

**5-S-TS1**

| | | | |
|---|---|---|---|
| Fe | 1.57043 | 0.07094 | 0.25033 |
| O | 0.70386 | 0.15685 | -1.22469 |
| C | 4.11108 | 0.07559 | -1.44038 |
| C | 5.02721 | -0.81274 | -2.1178 |
| C | 4.59705 | -2.08034 | -1.87512 |
| C | 3.42398 | -1.96626 | -1.03946 |
| N | 3.15088 | -0.65021 | -0.78342 |
| H | 5.87834 | -0.4877 | -2.70234 |
| H | 5.02215 | -3.01625 | -2.21438 |
| C | 1.55603 | 3.12168 | 0.19828 |
| C | 2.2595 | 4.26105 | -0.34389 |
| C | 3.33462 | 3.77033 | -1.01893 |
| C | 3.27972 | 2.33227 | -0.89764 |
| N | 2.1927 | 1.96253 | -0.15033 |
| H | 1.96008 | 5.29221 | -0.20623 |
| H | 4.10076 | 4.31342 | -1.55721 |
| C | -0.75633 | 0.0972 | 2.21398 |
| C | -1.65273 | 0.99053 | 2.90792 |
| C | -1.33886 | 2.2534 | 2.49883 |

| | | | |
|---|---|---|---|
| C | -0.23951 | 2.12879 | 1.57252 |
| N | 0.08561 | 0.80379 | 1.40128 |
| H | -2.41478 | 0.67557 | 3.60919 |
| H | -1.78405 | 3.19208 | 2.8032 |
| C | 1.62172 | -2.94758 | 0.32866 |
| C | 0.98658 | -4.0824 | 0.95393 |
| C | 0.0374 | -3.58915 | 1.793 |
| C | 0.07889 | -2.15336 | 1.67148 |
| N | 1.04743 | -1.7779 | 0.76846 |
| H | 1.24958 | -5.11467 | 0.7628 |
| H | -0.65606 | -4.12933 | 2.42331 |
| C | 2.7024 | -3.04088 | -0.53705 |
| C | -0.76762 | -1.2885 | 2.3435 |
| C | 0.41747 | 3.20392 | 0.99205 |
| C | 4.18453 | 1.45934 | -1.48595 |
| H | -1.49732 | -1.7245 | 3.01724 |
| H | 0.03404 | 4.19624 | 1.20958 |
| H | 5.00344 | 1.896 | -2.0494 |
| H | 3.03884 | -4.03757 | -0.80553 |
| S | 3.08613 | 0.20605 | 2.09203 |
| H | 3.2327 | -1.12851 | 2.24886 |
| H | -0.29615 | -0.49278 | -1.39237 |
| C | -5.16658 | -0.53967 | -1.4721 |
| C | -4.53327 | 0.30372 | -0.55526 |
| C | -5.34606 | 1.14401 | 0.21647 |
| C | -7.26322 | 0.39295 | -0.77018 |
| H | -4.8887 | 1.80352 | 0.95484 |
| H | -4.58607 | -1.23176 | -2.07534 |
| H | -8.34847 | 0.45244 | -0.83174 |
| C | -2.35611 | 1.35956 | -1.41629 |
| C | -1.91028 | 0.44314 | -2.57187 |
| H | -1.47139 | 1.80444 | -0.95368 |
| H | -3.03817 | 2.15506 | -1.7249 |
| C | -1.5004 | -0.81279 | -1.82574 |
| H | -2.74284 | 0.22702 | -3.25667 |
| H | -1.08425 | 0.86405 | -3.14885 |
| H | -1.34802 | -1.76514 | -2.32972 |
| N | -2.35444 | -0.88169 | -0.71586 |
| N | -2.62783 | -1.98492 | 0.01564 |
| O | -2.03356 | -2.9908 | -0.36774 |
| C | -3.02895 | 0.39249 | -0.40152 |
| H | -2.78851 | 0.677 | 0.62399 |
| C | -6.55503 | -0.49451 | -1.58297 |
| N | -6.67793 | 1.20232 | 0.12198 |

| | | | |
|---|---|---|---|
| H | -7.08082 | -1.1386 | -2.28153 |

**2-R-TS2**

| | | | |
|---|---|---|---|
| C | -1.91165 | 0.6438 | 0.94747 |
| C | -0.8573 | 0.04058 | 0.24237 |
| C | -1.18998 | -0.70544 | -0.89318 |
| C | -2.53012 | -0.81983 | -1.26433 |
| C | -3.49894 | -0.18838 | -0.48378 |
| N | -3.20301 | 0.53528 | 0.60718 |
| H | -0.42632 | -1.17416 | -1.50431 |
| H | -1.69016 | 1.24401 | 1.82527 |
| H | -2.8177 | -1.38123 | -2.1472 |
| H | -4.55189 | -0.25805 | -0.74607 |
| C | 0.54455 | 0.23525 | 0.81764 |
| C | 1.14755 | -0.98711 | 1.53521 |
| C | 1.60203 | -2.03118 | 0.49916 |
| H | 0.40789 | -1.4038 | 2.22588 |
| H | 1.99794 | -0.62195 | 2.12021 |
| C | 2.32469 | -1.26012 | -0.62214 |
| H | 2.265 | -2.77396 | 0.94834 |
| H | 0.74027 | -2.56914 | 0.09363 |
| H | 3.38767 | -1.11744 | -0.40942 |
| H | 2.22054 | -1.72833 | -1.60495 |
| N | 1.65349 | 0.05066 | -0.62471 |
| N | 2.19628 | 1.02856 | -1.17993 |
| O | 1.5803 | 2.19129 | -0.92309 |
| O | 0.80274 | 1.41417 | 1.28577 |
| H | 1.09018 | 2.07351 | 0.00869 |

**2-S-TS2**

| | | | |
|---|---|---|---|
| C | -1.18998 | 0.70523 | -0.89335 |
| C | -0.85727 | -0.04059 | 0.24232 |
| C | -1.9116 | -0.64367 | 0.94757 |
| C | -3.49894 | 0.18838 | -0.48372 |
| H | -1.69007 | -1.24377 | 1.82544 |
| H | -0.42629 | 1.17375 | -1.5046 |
| H | -4.5519 | 0.25808 | -0.74594 |
| C | 1.14753 | 0.9872 | 1.53515 |
| C | 1.60209 | 2.03125 | 0.49912 |
| H | 1.9979 | 0.62201 | 2.12015 |
| H | 0.40785 | 1.40386 | 2.22581 |
| C | 2.32458 | 1.26011 | -0.62224 |
| H | 0.74039 | 2.56935 | 0.09364 |
| H | 2.26519 | 2.77392 | 0.94828 |

| | | | |
|---|---|---|---|
| H | 2.22029 | 1.72828 | -1.60505 |
| H | 3.38759 | 1.11744 | -0.4097 |
| N | 1.65339 | -0.05068 | -0.62472 |
| N | 2.19624 | -1.02862 | -1.17981 |
| O | 1.5803 | -2.19134 | -0.92295 |
| C | 0.54461 | -0.23519 | 0.81752 |
| O | 0.80279 | -1.41406 | 1.28586 |
| H | 1.09012 | -2.07352 | 0.00879 |
| N | -3.20296 | -0.53513 | 0.60735 |
| C | -2.53014 | 0.81963 | -1.26443 |
| H | -2.81775 | 1.38088 | -2.14739 |

**5-R-TS2**

| | | | |
|---|---|---|---|
| C | 1.64571 | 0.043 | 1.07243 |
| C | 1.27789 | -0.23986 | -0.25169 |
| C | 2.28348 | -0.21329 | -1.22208 |
| C | 3.59232 | 0.0807 | -0.83795 |
| C | 3.84806 | 0.34751 | 0.50739 |
| N | 2.89537 | 0.33401 | 1.45425 |
| H | 2.0471 | -0.41841 | -2.2626 |
| H | 0.89646 | 0.04322 | 1.86172 |
| H | 4.39665 | 0.10845 | -1.56556 |
| H | 4.85521 | 0.58349 | 0.84205 |
| C | -0.14979 | -0.58379 | -0.62184 |
| C | -0.69882 | -1.90151 | -0.01935 |
| H | -0.23856 | -0.6153 | -1.71278 |
| C | -2.23249 | -1.78284 | -0.00092 |
| H | -0.31732 | -2.02652 | 0.99971 |
| H | -0.35683 | -2.75852 | -0.6036 |
| C | -2.59483 | -0.42488 | 0.59883 |
| H | -2.65454 | -1.8435 | -1.00983 |
| H | -2.68232 | -2.57362 | 0.60873 |
| H | -2.27527 | -0.32861 | 1.65274 |
| N | -1.13896 | 0.40512 | -0.12783 |
| N | -1.15974 | 1.56417 | -0.58284 |
| O | -2.21487 | 2.27953 | -0.15539 |
| O | -3.62594 | 0.23997 | 0.21678 |
| H | -2.94994 | 1.60002 | 0.14428 |

**5-S-TS2**

| | | | |
|---|---|---|---|
| C | 2.34673 | 0.61401 | -0.95952 |
| C | 1.42864 | -0.85194 | 0.69464 |
| C | 2.73436 | -1.09418 | 1.12538 |
| C | 3.78593 | -0.45053 | 0.47376 |
| N | 3.60368 | 0.39026 | -0.55791 |
| H | 0.59482 | -1.35001 | 1.17907 |
| H | 2.22981 | 1.29287 | -1.80272 |
| H | 2.93232 | -1.77226 | 1.94894 |
| H | 4.81453 | -0.61704 | 0.78387 |
| C | -0.66745 | 1.7888 | -0.49881 |
| C | -1.23026 | 1.66534 | 0.93462 |
| H | -1.46421 | 2.08305 | -1.18922 |
| H | 0.12818 | 2.53486 | -0.56158 |
| C | -2.02949 | 0.36465 | 1.07021 |
| H | -0.41319 | 1.65701 | 1.66298 |
| H | -1.88933 | 2.50589 | 1.16874 |
| H | -1.66403 | -0.31039 | 1.85972 |
| N | -1.21077 | -0.47052 | -0.35098 |
| N | -1.85884 | -1.21247 | -1.12436 |
| O | -2.97749 | -1.6938 | -0.57178 |
| O | -3.28807 | 0.32276 | 0.78777 |
| H | -3.29121 | -0.96779 | 0.15094 |
| C | -0.1566 | 0.3827 | -0.91842 |
| H | -0.15846 | 0.27722 | -2.00755 |
| C | 1.21593 | 0.02848 | -0.36991 |

**N1A-TS**

| | | | |
|---|---|---|---|
| N | 0.01965 | 3.66847 | -0.62144 |
| N | -0.30225 | 4.65497 | -1.0324 |
| N | 2.41125 | 0.20551 | 0.34488 |
| C | 2.47147 | -0.62023 | -0.74748 |
| N | 3.53659 | -1.28721 | -1.20355 |
| C | 4.64588 | -1.08281 | -0.45517 |
| C | 4.71449 | -0.27708 | 0.6934 |
| C | 3.53587 | 0.37438 | 1.10041 |
| N | 5.90954 | -1.60882 | -0.61973 |
| C | 6.67947 | -1.10226 | 0.42578 |
| N | 5.99532 | -0.30015 | 1.23662 |
| N | 3.49851 | 1.15921 | 2.20845 |
| C | 6.33155 | -2.52144 | -1.68687 |
| H | 1.54758 | -0.7465 | -1.29972 |
| H | 7.72103 | -1.35546 | 0.53973 |
| H | 4.33739 | 1.26345 | 2.76028 |

| | | | |
|---|---|---|---|
| H | 2.61867 | 1.43371 | 2.61244 |
| H | 6.72471 | -3.44746 | -1.26075 |
| H | 5.45667 | -2.74872 | -2.29722 |
| H | 7.09557 | -2.05229 | -2.31184 |
| C | -6.40375 | -1.9785 | 1.50715 |
| C | -7.43883 | -2.07683 | 0.57043 |
| N | -7.39649 | -1.47133 | -0.64038 |
| C | -6.30594 | -0.74518 | -0.94657 |
| C | -5.21193 | -0.58783 | -0.07065 |
| C | -5.2747 | -1.22442 | 1.1826 |
| C | -4.06276 | 0.23547 | -0.51303 |
| C | -2.85548 | 0.409 | 0.41204 |
| C | -1.74597 | 1.2158 | -0.27744 |
| C | -0.5666 | 1.52828 | 0.65847 |
| O | -4.0563 | 0.79829 | -1.6279 |
| C | 0.60305 | 2.19961 | -0.0298 |
| H | -6.48367 | -2.48092 | 2.46401 |
| H | -8.33252 | -2.65297 | 0.78227 |
| H | -6.29011 | -0.26813 | -1.9193 |
| H | -4.46328 | -1.13739 | 1.89727 |
| H | -3.18505 | 0.91088 | 1.33419 |
| H | -2.4822 | -0.57854 | 0.71621 |
| H | -1.39226 | 0.65873 | -1.15418 |
| H | -2.18772 | 2.14065 | -0.66948 |
| H | -0.88494 | 2.11371 | 1.52941 |
| H | -0.11609 | 0.60261 | 1.04178 |
| H | 1.42432 | 2.57184 | 0.58022 |
| H | 0.94262 | 1.76608 | -0.96899 |

**N3A-TS**

| | | | |
|---|---|---|---|
| N | -0.30947 | 3.76993 | -0.62937 |
| N | 0.00646 | 4.82621 | -0.68116 |
| N | -4.18444 | -0.84567 | -2.06695 |
| C | -3.17251 | -0.0776 | -1.67599 |
| N | -2.78768 | 0.25428 | -0.42848 |
| C | -3.58762 | -0.31444 | 0.50045 |
| C | -4.68406 | -1.14185 | 0.23656 |
| C | -4.97568 | -1.40195 | -1.12179 |
| N | -3.52166 | -0.23492 | 1.87362 |
| C | -4.57162 | -1.00792 | 2.34634 |
| N | -5.28623 | -1.56456 | 1.40216 |
| N | -6.00111 | -2.17564 | -1.50505 |
| C | -2.55921 | 0.50123 | 2.6733 |
| H | -2.5718 | 0.33681 | -2.48593 |

| | | | |
|---|---|---|---|
| H | -4.74473 | -1.11432 | 3.40936 |
| H | -6.60176 | -2.60173 | -0.81682 |
| H | -6.16432 | -2.33096 | -2.48766 |
| H | -2.71549 | 0.26071 | 3.72561 |
| H | -1.53866 | 0.21534 | 2.40302 |
| H | -2.68717 | 1.58143 | 2.54618 |
| C | 6.04379 | -2.25738 | -1.06453 |
| C | 7.08847 | -2.08673 | -0.15285 |
| N | 7.0653 | -1.19493 | 0.84822 |
| C | 5.97598 | -0.43602 | 0.96603 |
| C | 4.86416 | -0.51983 | 0.10765 |
| C | 4.91221 | -1.46003 | -0.93148 |
| C | 3.71164 | 0.38848 | 0.3508 |
| C | 2.49268 | 0.30984 | -0.57794 |
| C | 1.4182 | 1.30985 | -0.14628 |
| C | 0.18751 | 1.29491 | -1.0594 |
| O | 3.71097 | 1.19569 | 1.26909 |
| C | -0.93503 | 2.1832 | -0.59246 |
| H | 6.12098 | -2.9963 | -1.85484 |
| H | 7.98728 | -2.69473 | -0.2295 |
| H | 5.96083 | 0.2858 | 1.77807 |
| H | 4.08675 | -1.57301 | -1.62752 |
| H | 2.81628 | 0.50252 | -1.60949 |
| H | 2.09794 | -0.71444 | -0.57039 |
| H | 1.12106 | 1.09121 | 0.88529 |
| H | 1.86631 | 2.31002 | -0.11986 |
| H | 0.43805 | 1.52255 | -2.10112 |
| H | -0.28948 | 0.30657 | -1.05777 |
| H | -1.79073 | 2.34909 | -1.24158 |
| H | -1.20065 | 2.15245 | 0.46071 |

**N7A-TS**

| | | | |
|---|---|---|---|
| N | 0.3207 | -0.13829 | 0.08359 |
| N | 0.3193 | -0.16354 | 1.13786 |
| N | 0.56931 | -3.09816 | -6.40095 |
| C | 0.76615 | -2.34349 | -7.50559 |
| N | 0.9298 | -1.01557 | -7.58182 |
| C | 0.88209 | -0.41875 | -6.37753 |
| C | 0.68515 | -1.10832 | -5.19358 |
| C | 0.52555 | -2.47496 | -5.20547 |
| N | 1.01727 | 0.9237 | -6.14526 |
| C | 0.90544 | 1.05747 | -4.8424 |
| N | 0.70324 | -0.14669 | -4.22332 |
| N | 0.34426 | -3.12374 | -4.13357 |

| | | | |
|---|---|---|---|
| C | 1.23929 | 1.98523 | -7.13762 |
| H | 0.79565 | -2.88761 | -8.46113 |
| H | 0.96688 | 2.02014 | -4.31373 |
| H | 0.3135 | -2.61912 | -3.21328 |
| H | 0.22467 | -4.16643 | -4.16484 |
| H | 1.30445 | 2.96901 | -6.6211 |
| H | 2.18953 | 1.78957 | -7.68309 |
| H | 0.39244 | 2.00088 | -7.85967 |
| C | -7.52257 | 1.38046 | -1.08955 |
| C | -7.84419 | 2.71558 | -0.90416 |
| N | -6.93978 | 3.6706 | -0.59603 |
| C | -5.65608 | 3.26983 | -0.4681 |
| C | -5.23738 | 1.95931 | -0.63418 |
| C | -6.19213 | 0.99868 | -0.95103 |
| C | -3.93648 | 1.62364 | -0.49211 |
| C | -3.15162 | 1.12561 | -1.68082 |
| C | -1.72204 | 0.8195 | -1.25404 |
| C | -0.92989 | 0.31685 | -2.45378 |
| O | -3.40158 | 1.7222 | 0.58651 |
| C | 0.49968 | 0.01074 | -2.02701 |
| H | -8.29871 | 0.64218 | -1.3396 |
| H | -8.89688 | 3.01509 | -1.01427 |
| H | -4.89923 | 4.02773 | -0.21757 |
| H | -5.89812 | -0.05207 | -1.09064 |
| H | -3.62629 | 0.20002 | -2.07672 |
| H | -3.14277 | 1.90718 | -2.47319 |
| H | -1.24737 | 1.7451 | -0.85815 |
| H | -1.73088 | 0.03793 | -0.46168 |
| H | -1.34719 | -0.55599 | -3.00404 |
| H | -0.92106 | 1.09842 | -3.24615 |
| H | 0.79568 | -0.99917 | -1.8337 |
| H | 1.20804 | 0.80558 | -1.92048 |

**N3G-TS**

| | | | |
|---|---|---|---|
| N | 0.71365 | 3.11893 | -1.41868 |
| N | 0.58916 | 3.93967 | -2.16494 |
| N | 5.03853 | 0.64001 | 0.91956 |
| C | 3.73986 | 0.80845 | 1.31946 |
| N | 2.74773 | 0.00232 | 0.93462 |
| C | 3.17374 | -1.0685 | 0.17456 |
| C | 4.46655 | -1.3056 | -0.30343 |
| C | 5.5345 | -0.4121 | 0.04099 |
| N | 2.4036 | -2.12329 | -0.26856 |
| C | 3.26605 | -2.94992 | -1.00087 |
| N | 4.50304 | -2.48379 | -1.03895 |
| C | 0.99703 | -2.37978 | 0.03121 |
| H | 2.92319 | -3.86183 | -1.46155 |
| H | 0.79697 | -3.44548 | -0.09146 |
| H | 0.78592 | -2.10072 | 1.06592 |
| H | 0.3349 | -1.82381 | -0.64147 |
| C | -6.60984 | -0.54661 | 1.77238 |
| C | -7.53779 | -0.9873 | 0.82165 |
| N | -7.26215 | -1.04971 | -0.50294 |
| C | -6.03908 | -0.6682 | -0.91355 |
| C | -5.03751 | -0.20945 | -0.03285 |
| C | -5.34268 | -0.1523 | 1.33981 |
| C | -3.72605 | 0.18509 | -0.59499 |
| C | -2.62061 | 0.68405 | 0.33994 |
| C | -1.36569 | 1.07361 | -0.45462 |
| C | -0.21119 | 1.54614 | 0.4442 |
| O | -3.49336 | 0.11893 | -1.82027 |
| C | 1.07478 | 1.82731 | -0.28279 |
| H | -6.87711 | -0.51428 | 2.82207 |
| H | -8.53295 | -1.30199 | 1.11489 |
| H | -5.83775 | -0.72462 | -1.97675 |
| H | -4.61139 | 0.19253 | 2.06318 |
| H | -2.99489 | 1.53991 | 0.91941 |
| H | -2.38756 | -0.10325 | 1.07185 |
| H | -1.04859 | 0.21834 | -1.06352 |
| H | -1.6376 | 1.86052 | -1.17012 |
| H | -0.49924 | 2.40616 | 1.06033 |
| H | 0.08736 | 0.74744 | 1.14154 |
| H | 1.87567 | 2.33432 | 0.25681 |
| H | 1.41471 | 1.10145 | -1.01928 |
| O | 6.73133 | -0.41492 | -0.26293 |
| H | 5.7729 | 1.25109 | 1.2604 |
| N | 3.43604 | 1.89602 | 2.10906 |

| | | | |
|---|---|---|---|
| H | 2.60924 | 1.82841 | 2.68376 |
| H | 4.16303 | 2.51318 | 2.43884 |

**O6G-TS**

| | | | |
|---|---|---|---|
| N | 5.01988 | 1.04344 | 0.73802 |
| C | 3.99034 | 1.96247 | 0.72616 |
| H | 4.1379 | 2.97398 | 1.07927 |
| N | 2.86238 | 1.47592 | 0.25577 |
| C | 3.16468 | 0.16313 | -0.06012 |
| C | 2.3474 | -0.86704 | -0.60599 |
| O | 1.14953 | -0.82678 | -0.9317 |
| N | 3.07692 | -2.06409 | -0.7686 |
| H | 2.55023 | -2.82176 | -1.18985 |
| C | 4.40814 | -2.24358 | -0.44166 |
| N | 4.95377 | -3.4593 | -0.70554 |
| H | 4.38049 | -4.2768 | -0.84649 |
| H | 5.89441 | -3.60983 | -0.37006 |
| N | 5.15777 | -1.28869 | 0.06983 |
| C | 4.50017 | -0.1209 | 0.23224 |
| C | -5.45911 | -0.01058 | -0.84754 |
| C | -4.88984 | -0.40425 | 0.37472 |
| C | -5.7156 | -1.07018 | 1.29657 |
| C | -7.04287 | -1.30366 | 0.96036 |
| C | -7.50407 | -0.86535 | -0.2873 |
| N | -6.73383 | -0.23037 | -1.18156 |
| H | -5.30156 | -1.38643 | 2.24841 |
| H | -4.86833 | 0.50454 | -1.60191 |
| H | -7.71463 | -1.81322 | 1.64317 |
| H | -8.53736 | -1.03101 | -0.58253 |
| C | -3.46639 | -0.15067 | 0.72777 |
| C | -2.57472 | 0.60407 | -0.26782 |
| H | -3.04927 | 1.56601 | -0.50475 |
| H | -2.53514 | 0.03777 | -1.20739 |
| C | -1.16792 | 0.80076 | 0.29964 |
| H | -0.72306 | -0.17537 | 0.53699 |
| H | -1.18017 | 1.35124 | 1.24422 |
| C | -0.16049 | 1.37383 | -0.64393 |
| O | -2.99172 | -0.5291 | 1.78957 |
| N | -0.64956 | 3.02455 | -0.93709 |
| N | -0.84957 | 4.10179 | -1.07032 |
| H | -0.1725 | 1.04423 | -1.68023 |
| H | 0.8399 | 1.61948 | -0.26177 |
| C | 6.39398 | 1.25179 | 1.18925 |
| H | 6.64008 | 0.53502 | 1.97529 |

| | | | |
|---|---|---|---|
| H | 7.08738 | 1.12209 | 0.35535 |
| H | 6.4825 | 2.26532 | 1.58166 |

**N7G-TS**

| | | | |
|---|---|---|---|
| N | 3.62182 | 0.15251 | -1.597 |
| C | 4.26485 | -1.02509 | -1.26869 |
| N | 3.93212 | -1.76096 | -0.22823 |
| C | 2.91009 | -1.24398 | 0.48464 |
| C | 2.19016 | -0.07424 | 0.23905 |
| C | 2.54655 | 0.72942 | -0.88521 |
| N | 1.21138 | 0.1082 | 1.20003 |
| C | 1.34502 | -0.92869 | 1.99897 |
| N | 2.3573 | -1.78352 | 1.6194 |
| H | 3.94118 | 0.70471 | -2.38474 |
| N | 5.31072 | -1.3982 | -2.04831 |
| O | 2.05831 | 1.79836 | -1.27228 |
| H | 0.74532 | -1.12533 | 2.87739 |
| H | 5.42151 | -1.03848 | -2.98314 |
| H | 5.69807 | -2.3106 | -1.85908 |
| C | -3.09899 | -1.64604 | -0.0807 |
| C | -3.855 | -0.4836 | -0.29512 |
| C | -5.20391 | -0.63537 | -0.65387 |
| C | -5.72238 | -1.91688 | -0.77608 |
| C | -4.87616 | -3.00469 | -0.53639 |
| N | -3.58609 | -2.88347 | -0.19514 |
| H | -5.80803 | 0.24907 | -0.82627 |
| H | -2.048 | -1.58476 | 0.19585 |
| H | -6.75904 | -2.08018 | -1.05043 |
| H | -5.25212 | -4.02172 | -0.62283 |
| C | -3.29656 | 0.88937 | -0.16082 |
| C | -1.8209 | 1.03057 | 0.24835 |
| H | -1.18697 | 0.54671 | -0.50166 |
| H | -1.64163 | 0.49907 | 1.18681 |
| C | -1.4718 | 2.53244 | 0.38099 |
| H | -2.09776 | 2.97956 | 1.1562 |
| H | -1.7042 | 3.01535 | -0.5714 |
| C | -0.02418 | 2.62886 | 0.69786 |
| H | 0.72558 | 2.38969 | -0.07372 |
| H | 0.31402 | 2.48559 | 1.71982 |
| O | -3.97137 | 1.88404 | -0.36537 |
| N | 0.33585 | 4.45513 | 0.65936 |
| N | 0.58968 | 5.52078 | 0.52674 |
| C | 2.76585 | -3.02478 | 2.27276 |
| H | 3.00262 | -2.83836 | 3.3224 |

| | | | |
|---|---|---|---|
| H | 1.97357 | -3.77387 | 2.20195 |
| H | 3.65509 | -3.39265 | 1.76165 |

**N3C-TS**

| | | | |
|---|---|---|---|
| N | 0. | 0. | 0. |
| C | 0. | 0. | 1.3639 |
| H | 0.97872 | 0. | 1.83384 |
| C | -1.16621 | -0.01094 | 2.06536 |
| H | -1.16603 | -0.01044 | 3.14794 |
| C | -2.37768 | -0.04128 | 1.30788 |
| N | -3.57231 | -0.07481 | 1.93975 |
| H | -4.4232 | -0.04724 | 1.40009 |
| H | -3.63719 | -0.03836 | 2.94441 |
| N | -2.38303 | -0.04484 | -0.02719 |
| C | -1.20868 | -0.0076 | -0.71843 |
| O | -1.17996 | 0.02428 | -1.96542 |
| C | -3.0112 | 3.3427 | -6.02258 |
| C | -3.437 | 4.08347 | -4.90769 |
| C | -3.47677 | 5.48305 | -5.02906 |
| C | -3.09865 | 6.06238 | -6.23346 |
| C | -2.69287 | 5.22514 | -7.28031 |
| N | -2.64636 | 3.88913 | -7.18591 |
| H | -3.80111 | 6.08012 | -4.183 |
| H | -2.95541 | 2.25694 | -5.98721 |
| H | -3.11519 | 7.13871 | -6.36929 |
| H | -2.39171 | 5.64495 | -8.23706 |
| C | -3.84288 | 3.45542 | -3.62025 |
| C | -3.82942 | 1.9256 | -3.5016 |
| H | -2.82525 | 1.55489 | -3.74242 |
| H | -4.50221 | 1.51701 | -4.26906 |
| C | -4.23867 | 1.47999 | -2.09659 |
| H | -5.20761 | 1.89609 | -1.80723 |
| H | -3.5203 | 1.85178 | -1.35805 |
| C | -4.20096 | -0.00919 | -1.86412 |
| O | -4.19139 | 4.12934 | -2.66079 |
| N | -5.47374 | -0.6514 | -2.73949 |
| N | -6.35619 | -1.10107 | -3.22782 |
| H | -4.47533 | -0.37548 | -0.86789 |
| H | -3.66348 | -0.62618 | -2.58061 |
| C | 1.2786 | -0.03632 | -0.7244 |
| H | 1.31998 | -0.9168 | -1.33099 |
| H | 1.36047 | 0.82996 | -1.3471 |
| H | 2.08603 | -0.04856 | -0.0224 |

**O2C-TS**

| | | | |
|---|---|---|---|
| N | 3.62182 | 0.15251 | -1.597 |
| C | 4.26485 | -1.02509 | -1.26869 |
| N | 3.93212 | -1.76096 | -0.22823 |
| C | 2.91009 | -1.24398 | 0.48464 |
| C | 2.19016 | -0.07424 | 0.23905 |
| C | 2.54655 | 0.72942 | -0.88521 |
| N | 1.21138 | 0.1082 | 1.20003 |
| C | 1.34502 | -0.92869 | 1.99897 |
| N | 2.3573 | -1.78352 | 1.6194 |
| H | 3.94118 | 0.70471 | -2.38474 |
| N | 5.31072 | -1.3982 | -2.04831 |
| O | 2.05831 | 1.79836 | -1.27228 |
| H | 0.74532 | -1.12533 | 2.87739 |
| H | 5.42151 | -1.03848 | -2.98314 |
| H | 5.69807 | -2.3106 | -1.85908 |
| C | -3.09899 | -1.64604 | -0.0807 |
| C | -3.855 | -0.4836 | -0.29512 |
| C | -5.20391 | -0.63537 | -0.65387 |
| C | -5.72238 | -1.91688 | -0.77608 |
| C | -4.87616 | -3.00469 | -0.53639 |
| N | -3.58609 | -2.88347 | -0.19514 |
| H | -5.80803 | 0.24907 | -0.82627 |
| H | -2.048 | -1.58476 | 0.19585 |
| H | -6.75904 | -2.08018 | -1.05043 |
| H | -5.25212 | -4.02172 | -0.62283 |
| C | -3.29656 | 0.88937 | -0.16082 |
| C | -1.8209 | 1.03057 | 0.24835 |
| H | -1.18697 | 0.54671 | -0.50166 |
| H | -1.64163 | 0.49907 | 1.18681 |
| C | -1.4718 | 2.53244 | 0.38099 |
| H | -2.09776 | 2.97956 | 1.1562 |
| H | -1.7042 | 3.01535 | -0.5714 |
| C | -0.02418 | 2.62886 | 0.69786 |
| H | 0.72558 | 2.38969 | -0.07372 |
| H | 0.31402 | 2.48559 | 1.71982 |
| O | -3.97137 | 1.88404 | -0.36537 |
| N | 0.33585 | 4.45513 | 0.65936 |
| N | 0.58968 | 5.52078 | 0.52674 |
| C | 2.76585 | -3.02478 | 2.27276 |
| H | 3.00262 | -2.83836 | 3.3224 |
| H | 1.97357 | -3.77387 | 2.20195 |
| H | 3.65509 | -3.39265 | 1.76165 |

**O2T-TS**

| | | | |
|---|---|---|---|
| N | -3.60125 | 0.0466 | 1.38879 |
| C | -4.78484 | -0.68308 | 1.30698 |
| H | -5.27053 | -0.85538 | 2.26073 |
| C | -5.31055 | -1.15192 | 0.15107 |
| C | -6.58726 | -1.93755 | 0.07239 |
| H | -7.02656 | -2.07952 | 1.0628 |
| H | -7.31568 | -1.42716 | -0.56581 |
| H | -6.40864 | -2.91943 | -0.37742 |
| C | -4.59006 | -0.87547 | -1.09214 |
| O | -4.92024 | -1.22097 | -2.21394 |
| N | -3.39969 | -0.12848 | -0.91073 |
| H | -2.8784 | 0.06757 | -1.75754 |
| C | -2.87415 | 0.34655 | 0.26218 |
| O | -1.81578 | 1.00161 | 0.30587 |
| C | 5.01035 | -0.13967 | 0.6604 |
| C | 4.12958 | -0.91855 | -0.10918 |
| C | 4.54388 | -2.21342 | -0.46958 |
| C | 5.79415 | -2.65423 | -0.05696 |
| C | 6.59009 | -1.7909 | 0.70711 |
| N | 6.21212 | -0.55594 | 1.06606 |
| H | 3.8833 | -2.83909 | -1.06079 |
| H | 4.74307 | 0.86688 | 0.97514 |
| H | 6.15454 | -3.64423 | -0.31574 |
| H | 7.57428 | -2.10412 | 1.04663 |
| C | 2.79346 | -0.44025 | -0.54143 |
| C | 2.35169 | 0.9916 | -0.18224 |
| H | 3.16004 | 1.69947 | -0.3967 |
| H | 2.17746 | 1.02696 | 0.90197 |
| C | 1.08074 | 1.35441 | -0.95307 |
| H | 0.39112 | 0.49065 | -0.9192 |
| H | 1.2715 | 1.5277 | -2.01594 |
| C | 0.22347 | 2.40871 | -0.37217 |
| O | 2.01916 | -1.15471 | -1.16342 |
| N | 1.17456 | 3.93985 | -0.41191 |
| N | 1.77215 | 4.86511 | -0.47942 |
| H | -0.61154 | 2.78792 | -0.95538 |
| H | 0.04148 | 2.41264 | 0.69967 |
| C | -3.09407 | 0.52075 | 2.68193 |
| H | -2.09733 | 0.11552 | 2.86904 |
| H | -3.04488 | 1.61201 | 2.69497 |
| H | -3.7725 | 0.1823 | 3.46416 |

**O4T-TS**

| | | | |
|---|---|---|---|
| N | 5.00502 | -1.2497 | -0.28152 |
| C | 4.03222 | -1.67122 | 0.58919 |
| H | 4.22734 | -2.62955 | 1.05849 |
| C | 2.89763 | -0.97361 | 0.87065 |
| C | 1.85294 | -1.4569 | 1.83686 |
| H | 2.11488 | -2.43939 | 2.23645 |
| H | 1.74888 | -0.76703 | 2.68149 |
| H | 0.86921 | -1.53345 | 1.36248 |
| C | 2.70907 | 0.29912 | 0.20308 |
| O | 1.72568 | 1.04444 | 0.35397 |
| N | 3.73112 | 0.66621 | -0.65914 |
| H | 3.64221 | 1.55239 | -1.14121 |
| C | 4.89934 | -0.03558 | -0.95748 |
| O | 5.7375 | 0.37761 | -1.7335 |
| C | -4.76078 | -0.18282 | -0.94024 |
| C | -3.90011 | -0.85578 | -0.05836 |
| C | -4.25367 | -2.15767 | 0.33535 |
| C | -5.42858 | -2.70853 | -0.15473 |
| C | -6.21099 | -1.94343 | -1.02713 |
| N | -5.89024 | -0.70395 | -1.42141 |
| H | -3.60359 | -2.70056 | 1.01311 |
| H | -4.53308 | 0.8237 | -1.28625 |
| H | -5.74141 | -3.70828 | 0.12645 |
| H | -7.13853 | -2.3452 | -1.42861 |
| C | -2.64329 | -0.2653 | 0.4605 |
| C | -2.27379 | 1.17999 | 0.07369 |
| H | -3.15168 | 1.83008 | 0.14615 |
| H | -1.97567 | 1.17052 | -0.98396 |
| C | -1.13274 | 1.67896 | 0.96111 |
| H | -0.42571 | 0.84521 | 1.12905 |
| H | -1.47344 | 1.9817 | 1.95492 |
| C | -0.20738 | 2.67318 | 0.37589 |
| O | -1.8784 | -0.89915 | 1.17372 |
| N | -1.1265 | 4.16169 | 0.11327 |
| N | -1.75126 | 5.0622 | -0.01546 |
| H | 0.55635 | 3.10653 | 1.01668 |
| H | 0.12123 | 2.53824 | -0.65121 |
| C | 6.21415 | -2.03991 | -0.54953 |
| H | 6.16137 | -2.96494 | 0.02369 |
| H | 6.28073 | -2.26692 | -1.61489 |
| H | 7.10156 | -1.47428 | -0.2596 |
